# G protein-coupled receptor 107 deficiency promotes development of diabetic nephropathy

**DOI:** 10.1186/s43556-025-00250-1

**Published:** 2025-02-11

**Authors:** Deping Xu, Ziwen Tong, Ping Yang, Qiong Chen, Suhua Wang, Wei Zhao, Linzi Han, Yu Yin, Ruyue Xu, Min Zhang, Chunlin Cai, Deguang Wang, Dandan Zang, Guoling Zhou, Haisheng Zhou

**Affiliations:** 1 Department of Biochemistry and Molecular Biology, Anhui Medical University, Hefei, China; 2 The Clinical Laboratory, Hefei Affiliated Hospital to Anhui Medical University, the Second People’s Hospital of Hefei, Hefei, China; 3 Department of Pathology, the First Affiliated Hospital of Anhui Medical University, Hefei, China; 4 The Clinical Laboratory, the First Affiliated Hospital of Anhui Medical University, Hefei, China; 5 Department of Pathophysiology, Anhui Medical University, Hefei, China; 6 Department of Nephrology, the Second Affiliated Hospital of Anhui Medical University, Hefei, China; 7 Center for Scientific Research, Anhui Medical University, Hefei, China; 8 Center for Computational Integrative Biology (CCIB), Massachusetts General Hospital (MGH), Harvard Medical Colleague, Boston, MA USA

**Keywords:** Diabetic nephropathy, GPR107, Collagen type IV, Endocytosis, Podocytes

## Abstract

Diabetic nephropathy (DN) is characterized by glomerular basement membrane (GBM) thickening, primarily due to the abnormal accumulation of collagen type IV (COL4) in the extracellular matrix (ECM) of podocytes. Podocytes endocytosis is crucial for maintaining COL4 balance and GBM integrity. Previous studies have shown that G protein-coupled receptor 107 (GPR107) facilitates clathrin-dependent transferrin internalization and recycling in murine embryonic fibroblast cells. Therefore, the aim of the study is to investigate the role of GPR107 in regulating COL4 balance within the podocytes ECM and its potential as a therapeutic target for DN. Here, we found a significant decrease in GPR107 expression in renal tissues from DN patients and streptozocin (STZ)-induced DN mice. Furthermore, GPR107-deficient mice with STZ-induced DN exhibited more severe kidney damage, marked by increased GBM thickening and COL4 accumulation. In vitro, GPR107 deficiency under high-glucose conditions promoted COL4 accumulation in the ECM of podocytes due to increased COL4 production and decreased COL4 degradation. Mechanistically, we demonstrated that GPR107 contributes to angiotensin II receptor type 1 (AT1R) internalization through clathrin-mediated endocytosis (CME) in podocytes. Therefore, GPR107 deficiency impairs AT1R internalization, leading to increased membrane-bound AT1R. This, in turn, activates the AT1R/Ca^2+^ signaling pathway to promote phosphorylation of cAMP-response element-binding protein (CREB), ultimately enhancing COL4 synthesis and inhibiting the expression of matrix metalloproteinase 2 (MMP-2). These findings shed light on new functions of GPR107 in DN and offer new insights into a therapeutic target for DN.

**Supplementary Information:**

The online version contains supplementary material available at 10.1186/s43556-025-00250-1.

## Introduction

Diabetic nephropathy (DN) is a prevalent complication of both type I and type II diabetes mellitus (T1DM and T2DM), and the leading cause of end-stage renal disease globally [1, 2]. Current therapeutic strategies emphasize tight blood glucose control, renin–angiotensin–aldosterone system blockade and the use of SGLT2 inhibitors but the mechanisms contributing to DN remain obscure and definitive treatments have yet to be developed [3].

Clinically, DN is characterized by progressive proteinuria subsequent to destruction of the glomerular filtration barrier [4, 5]. Morphologically, DN features expansion of the mesangial matrix, glomerular hypertrophy, and thickening of glomerular basement membranes (GBM) [6]. Thickening of the GBM is believed to be due to changes in the metabolism of extracellular matrix (ECM), primarily in podocytes and endothelial cells [7, 8]. However, a detailed accounting of the imbalance between ECM synthesis and degradation that results in accumulation of ECM components and subsequent renal compromise has not been established [9, 10].

Collagen type IV (COL4) is the main component of GBM [11]. Both T1DM and T2DM are associated with abnormal accumulation of COL4 in the GBM due to a reduction in degradation and an excessive production of COL4 by podocytes [12, 13]. Impairment or depletion of podocytes functions significantly contribute to DN. For example, damage to the podocyte's endocytosis pathway, regulated by angiotensin II (Ang-II), contributes to the development of proteinuria in DN due to impaired glomerular filtration barrier integrity [14–16]. Inhibition of the internalization of AT1R in podocytes, which depends on clathrin-mediated endocytosis (CME) [14, 17–19], could elevate calcium concentration induced by Ang-II signaling, activates the renin–angiotensin–aldosterone system (RAAS) and exacerbates kidney injury, including increased proteinuria, glomerular sclerosis, and abnormal basement membrane thickness [14, 20–22].

G protein-coupled receptor 107 (GPR107), an orphan receptor, was initially identified among cDNAs enriched for expression in human lung. It is expressed in various tissues, including the brain, heart, liver, and kidney [23, 24]. We previously demonstrated that GPR107 interacts directly or indirectly with a limited subset of cellular surface receptors and participates in internalization and recycling of extracellular transferrin through the CME pathway [25]. Therefore, our hypothesis is that GPR107 interacts with AT1R in podocytes and is involved in podocytes endocytosis to maintain the balance of ECM components and the integrity of the GBM. To test this hypothesis, we used mouse models and cellular models with genetically engineered loss-of-function mutations in GPR107 in the present study. We demonstrated a significant decrease of GPR107 expression in renal tissues from both DN patients and streptozocin (STZ)-induced DN mice. The GPR107-deficient mice with DN exhibit aggravated nephropathy, which characterized by the presence of a thickened GBM mainly resulting from an accumulation of COL4.

## Results

### GPR107 deficiency exacerbates DN by increasing the thickness of the GBM due to excessive deposition of COL4

**Fig. 1:**
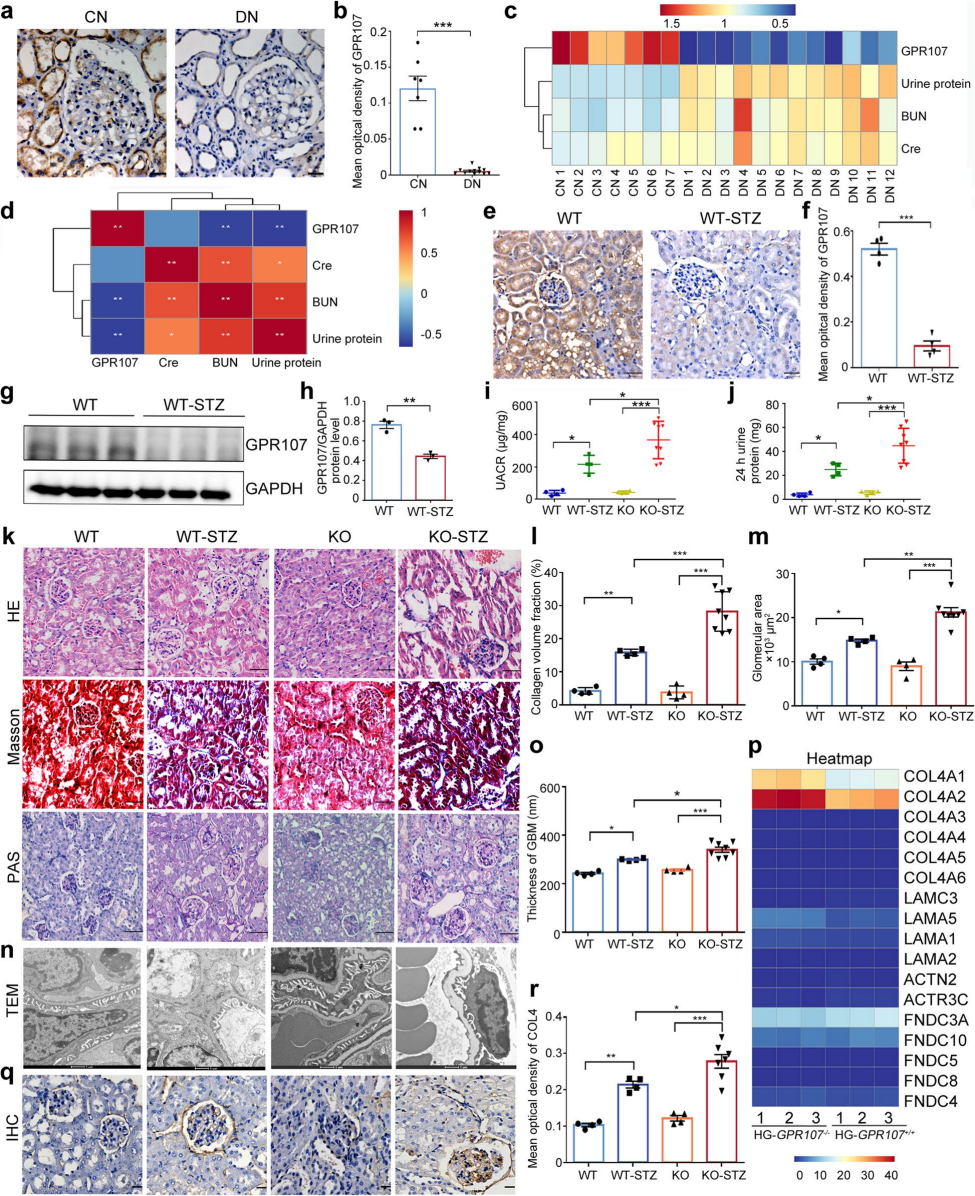
GPR107 deficiency exacerbates DN characterized by an increase in the thickness of GBM and excessive deposition of COL4. **a**, **b** Immunostaining of GPR107 was performed on nephrectomy specimens from healthy donors, and kidney biopsies obtained from patients with DN. Original magnification × 400, scale bar = 20 μm. *** *P* < 0.001. **c**, **d** The expression level of GPR107, 24-h urinary protein, blood urea nitrogen and creatinine levels in DN patients and healthy controls, and correlation analysis between the two factors. * *P* < 0.05, ** *P* < 0.01. **e**, **f** Immunostaining analysis for GPR107 expression in the kidneys of mice. Original magnification × 400, bar = 20 μm. *** *P* < 0.001. **g**, **h** Western blotting analysis of GPR107 in the WT-STZ mice and WT mice. The data represent gray scales from three independent experiments. ** *P* < 0.01. **i**, **j** Kidney function analysis of mice at 12 weeks after induction of diabetes with STZ. * *P* < 0.05, *** *P* < 0.001. **k** Representative images of the H&E staining, Masson’s trichrome-stained, and periodic acid-Schiff (PAS) in mice. Original magnification × 400, bar = 50 μm. **l** Dot plots were generated for summarizing the fraction of collagen volume fraction from Masson’s trichrome-stained sections. **m** Dot plots were created for summarizing glomerular area from the PAS sections. * P < 0.05, ** *P* < 0.01, *** P < 0.001. **n**, **o** Representative images and GBM thickness of transmission electronic microscopy. Original magnification × 6400, bar = 1 μm. * *P* < 0.05, *** *P* < 0.001. **p** Heatmap of up- and down-regulated 17 genes associated with the ECM of GBM in *GPR107*^+*/*+^ and *GPR107*.^*−/−*^ HPC incubated with HG for 5 days. **q**, **r** Immunostaining for COL4 protein in kidney tissues. * *P* < 0.05, ** *P* < 0.01, *** *P* < 0.001. Original magnification × 400, bar = 20 μm. Each dot represents data obtained from 1 mouse specimen. Data shown as the mean ± SE for each group. BUN, blood urea nitrogen. Cre, creatinine. WT, wild type. KO, knockout. STZ, streptozotocin. GBM, glomerular basement membranes. WT, KO, and WT-STZ mice, *n* = 4. KO-STZ mice, *n* = 8

To investigate the roles of GPR107 in DN, we first performed immunohistochemical analyses to detect the expression of GPR107 in renal tissue samples obtained from kidney biopsies of patients with DN and from nephrectomy samples containing healthy renal parenchyma. The results showed that GPR107 is mainly expressed in both the renal medulla and renal cortex. Interestingly, kidney biopsies from healthy controls showed more intense staining of GPR107 than DN patients (Fig. 1a, b). Renal GPR107 expression, 24-h urinary protein, blood urea nitrogen (BUN) and creatinine were statistically analyzed in DN patients and healthy controls. The results showed that the expression level of GPR107 in DN group was low, accompanied by renal function impairment and a large amount of 24-h urinary protein.There was a significant negative correlation between the level of GPR107 and 24-h urinary protein and urea nitrogen, that is, the lower the expression level of GPR107, the more severe the renal function injury and the more urinary protein (Fig. 1c, d). In order to further identify expression patterns of GPR107 in DN, we established mouse models of DN by using STZ. IHC analysis showed that GPR107 was predominantly expressed in both the renal medulla and renal cortex of WT mice, similar to its expression pattern in various renal cells, including HPC, mesangial cells (MCs), proximal tubular epithelial cells (HK-2), distal tubular epithelial cells (HKC), and primary cultured tubular epithelial cells (TECs) (Fig. S1a, b). However, a noticeable decrease in GPR107 expression was histochemically observed in renal tissues of mice with STZ-induced DN when compared to the control group (Fig. 1e, f). The expression of GPR107 was also found by immunoblotting to be markedly reduced in kidney tissues of mice with STZ-induced DN, as compared to normal mice (Fig. 1g, h). The findings suggested that deficiency of GPR107 might contribute to the progression of DN. Consequently, we attempted to establish DN models utilizing *Gpr107*-null mice, aiming to further explore its functions in DN. Generation and identification of conditional knock out *Gpr107* mice is described in the supplementary results (Fig. S1c-h). The *Gpr107*^*hgfp_flox*^ (pre-induction) mice and *Gpr107*^*cko*^ (post-induction) mice are described as wild type (WT) mice and knockout (KO) mice, respectively.

We created models of STZ-induced DN in both *Gpr107*^+/+^ (wild-type, WT) and *Gpr107*^cko^ (knockout, KO) mice at the age of 8 weeks by administering STZ. In both WT and KO mice, the blood glucose levels in the STZ-treated mice significantly increased, whereas the body weight of the STZ-treated mice notably decreased compared to that of the normal mice. There was no significant difference observed in blood glucose levels and body weight between WT-STZ mice and KO-STZ mice (Fig. S2a, 2b). Diabetic WT mice displayed increased levels of UACR and 24 h urine protein, which was further increased significantly in diabetic *Gpr107* KO mice (Fig. 1i, j). Consistent with impaired kidney function, renal histological analyses, including H&E staining, Masson’s trichrome staining, and periodic acid-Schiff (PAS) staining, were more intense in diabetic *Gpr107* KO mice than diabetic WT mice (Fig. 1k). KO-STZ mice exhibited a significant increase in collagen volume fraction and glomerular size in renal tissue compared to WT-STZ mice (Fig. 1l, m). An increased thickness of the GBM was observed by ultrastructural analysis of the kidneys of both WT-STZ mice and KO-STZ mice in comparison to normal mice (Fig. 1n, Fig. S2c), and KO-STZ mice exhibited a more pronounced thickening of the GBM compared to WT-STZ mice. The GBM thickness was significantly increased in the STZ-treated groups compared to controls. In the WT-STZ group, GBM thickness was 299.8 ± 2.529 nm, compared to 242.5 ± 4.173 nm in the WT group (*P* = 0.0054). Similarly, KO-STZ animals exhibited a significantly increased GBM thickness (340.1 ± 10.43 nm) compared to the KO control group ( 256 ± 4.628 nm) (*P* < 0.0001) (Fig. 1o). These findings suggest that a deficiency in GPR107 aggravates DN, and this is correlated with an increase in the thickness of the GBM.

Given the crucial role of podocytes in maintaining the integrity and function of the GBM, we explored the involvement of GPR107 in podocytes. A *GPR107*^−/−^ HPC clone was successfully generated by utilizing the CRISPR-Cas9 technique (Fig. S2d-2f). A transcriptomic analysis was conducted to identify differences in the ECM of *GPR107*^−/−^ and *GPR107*^+/+^ HPC cultured for 5 days in a medium with high glucose (HG). We found a notable upregulation of *COL4A* expression in *GPR107*^−/−^ HPC when compared to *GPR107*^+/+^ HPC, among the 17 genes associated with the ECM of GBM (Fig. 1p). These findings were corroborated by a significant increase in the level of COL4 in the GBM of KO-STZ mice when compared to WT-STZ mice (Fig. 1q, r).

### Deficiency of GPR107 decreases COL4 endocytosis of podocytes under high glucose conditions

**Fig. 2:**
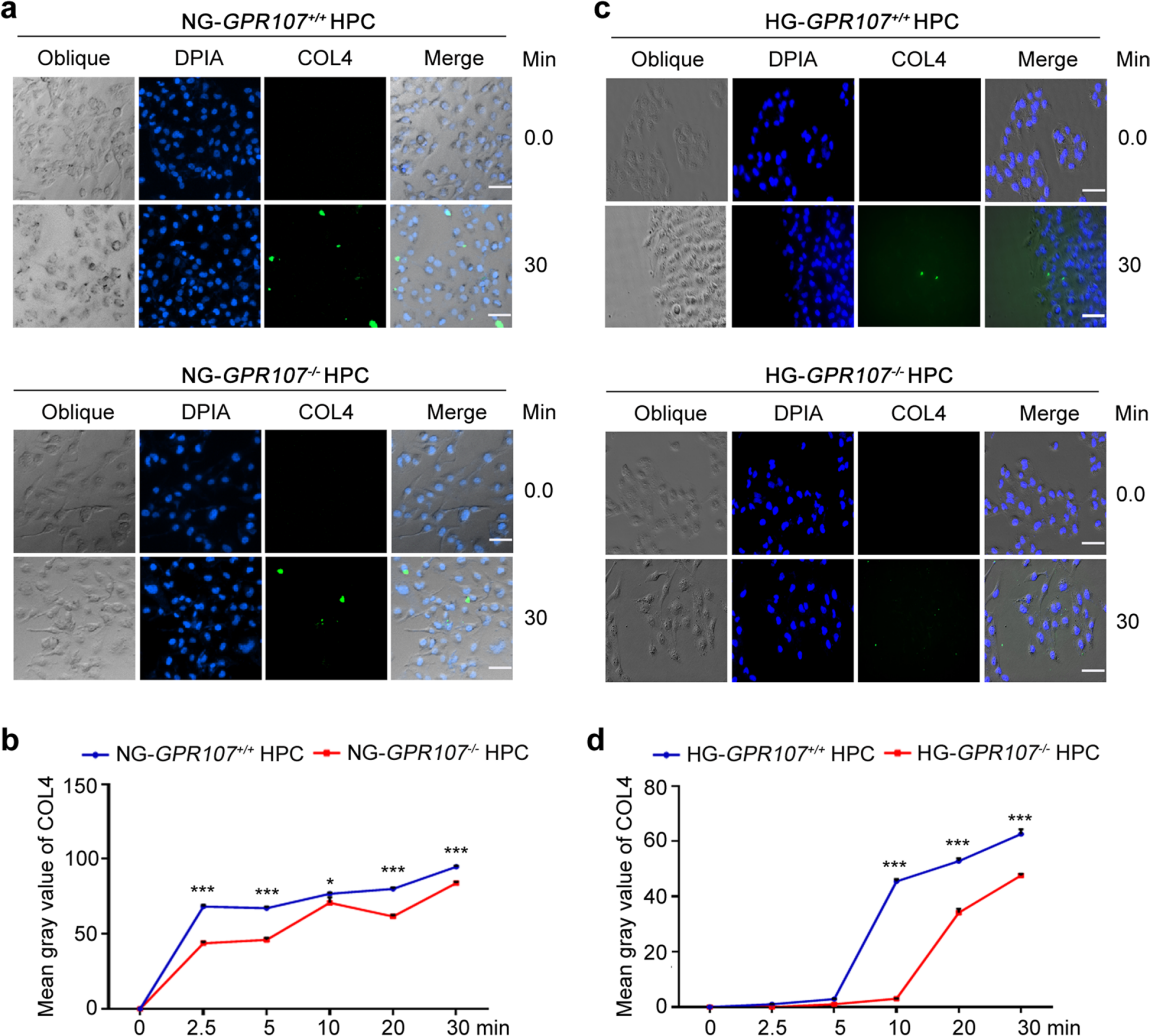
The deficiency of GPR107 decreases the endocytosis of COL4 in podocytes under high glucose conditions. Both *GPR107*^+*/*+^ and *GPR107*^*−/−*^ HPC cells were co-cultured with the quenched fluorescent substrate DQ-COL4 under different glucose concentrations for 30 min at 37℃. **a**, **b** The cells were incubated under NG conditions and then subjected to quantitative analysis of the intensity of green fluorescence within the cells. Original magnification × 200, bar = 20 μm. ** *P* < 0.01, *** *P* < 0.001 when compared with *GPR107*^+*/*+^ HPC. **c**, **d** The cells were incubated under HG conditions and subsequently subjected to quantitative analysis in order to assess the intensity of green fluorescence within them. Original magnification × 200, bar = 20 μm. *** *P* < 0.001 compared with *GPR107*.^+*/*+^ HPC. Results are expressed as the mean ± SE of three samples from each group. HG, high glucose (20 mM). NG, normal glucose (11.1 mM)

As GPR107 is involved in CME [25], we hypothesized that an increase of COL4 in the GBM would be associated with a diminished endocytosis of podocytes due to GPR107 deficiency. Endocytosis analysis of COL4 was producted to verify. We found the cytoplasm of both *GPR107*^+/+^ and *GPR107*^−/−^ HPC exhibited green fluorescent degradation products subsequent to internalizing dye-quenched COL4 (DQ-COL4), after 2.5 min of co-culture in NG. These signals gradually became stronger at 5 to 30 min (Fig. 2a, b and Fig. S3a). The signals initially displayed by *GPR107*^+/+^ and *GPR107*^−/−^ HPC cells were observed after culturing them in HG for 10 and 20 min, respectively (Fig. 2c, d and Fig. S3b). Both *GPR107*^+/+^ and *GPR107*^−/−^ HPC in HG displayed weaker signals in comparison to cells incubated in NG. A reduction in the intensity of green fluorescence was observed in the *GPR107*^−/−^ HPC in HG compared to *GPR107*^+/+^ HPC in HG. *GPR107*^−/−^ HPC transfected with GPR107 cDNA plasmid were rescued and called RC HPC. Western blotting experiment proved that the rescue was successful (Fig. S3c). DQ-COL4 was incubated in *GPR107*^+*/*+^, *GPR107*^*−/−*^ and RC HPC under NG and HG conditions. It was found that under NG and HG conditions, the reduction of COL4 endocytosis by *GPR107*^*−/−*^ HPC was recovered in RC HPC (Fig. S4a, 4b). The findings indicate that a deficiency in GPR107 decreases the endocytosis of COL4 in podocytes in HG. This reduction in endocytosis at least partially contributes to an increase in COL4 in the GBM of DN.

### GPR107 is involved in the endocytosis of AT1R in podocytes through CME, influencing the levels of AT1R in podocytes

**Fig. 3:**
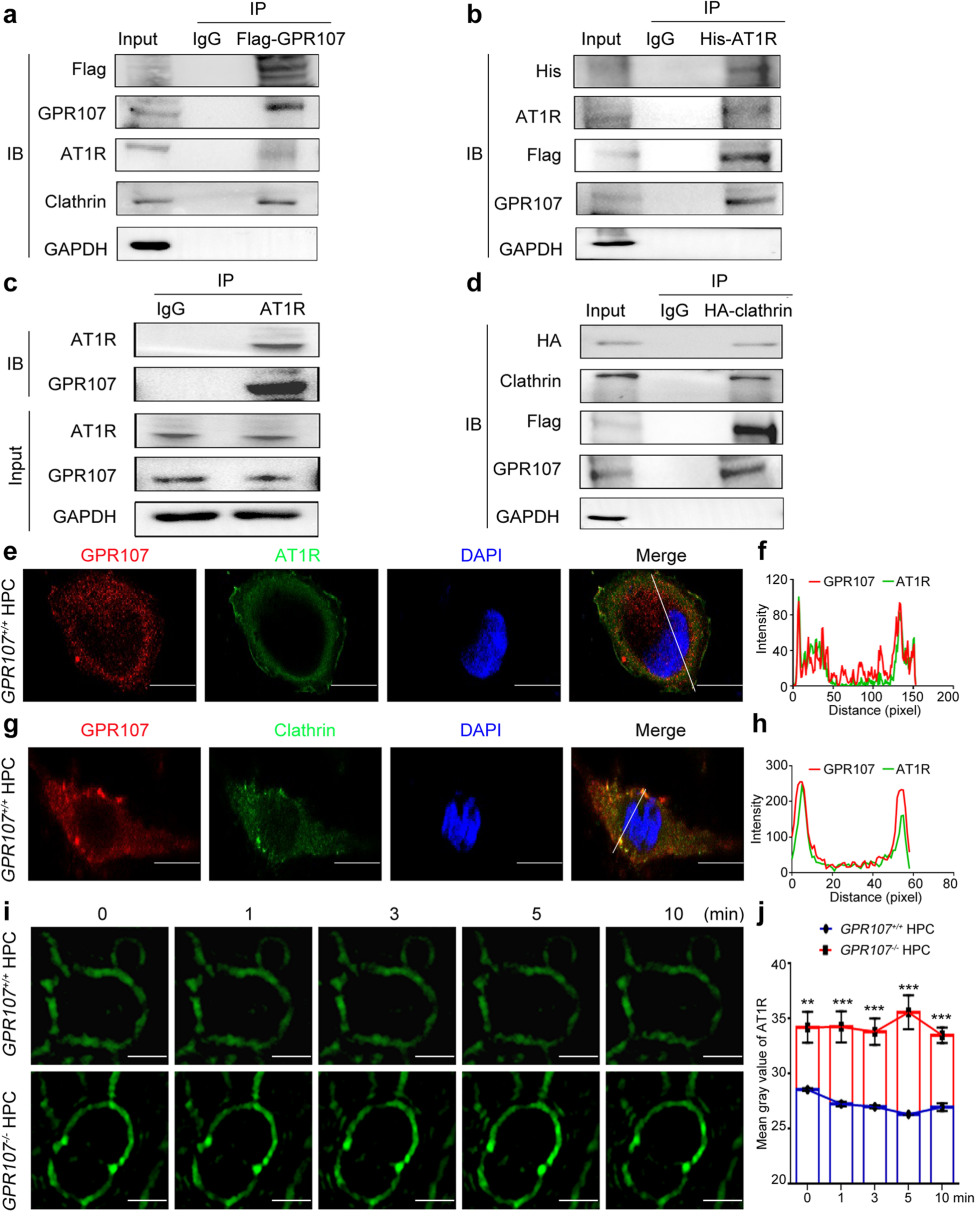
GPR107 is involved in the endocytosis of AT1R in podocytes through CME. **a**, **b** Co-immunoprecipitation (Co-IP) analysis for interactions between GPR107 and AT1R in HPC. **c** The proteins were extracted and immunoprecipitated using AT1R antibody to validate endogenous interaction. **d** Co-IP and Western blotting analysis were performed to examine the interaction between the clathrin and GPR107 in HPC. **e**, **f** Double immunofluorescent and analysis for labeling of GPR107 (red) and AT1R (green) in the HPC. Original magnification × 640, bar = 10 μm. **g**, **h** Double immunofluorescent and analysis for GPR107 (red) and clathrin (green) in HPC. Original magnification × 640, bar = 10 μm. **i**, **j** Analyzing the distribution of AT1R on the cell membrane. Cells were transfected with the GFP-tagged AT1R and then treated with Ang-II (100 μM) for 1, 3, 5, and 10 min. ** *P* < 0.01, *** *P* < 0.001 compared to the *GPR107*^+*/*+^ HPC. Original magnification × 200, bar = 10 μm. AT1R, Angiotensin II type1 receptor. Ang-II, Angiotensin II

Under hyperglycemic conditions, podocytes usually exhibit enhanced recruitment and internalization of membrane-bound receptors, particularly the AT1R, through the clathrin-mediated pathway [18]. To avoid over-activation of the RAAS, through endocytosis of AT1R on the cell membrane, podocytes can reduce its expression on the cell membrane, thereby reducing its binding to Ang-II and reducing the damage of RAAS to the kidney. Consequently, we aimed to investigate the interaction and correlation in endocytosis between GPR107 and AT1R. Co-IP of proteins expressed in HPC cells were performed using Flag-tagged GPR107. Subsequent immunoblotting analysis showed the presence of endogenous AT1R and clathrin in the HPC (Fig. 3a). Both Flag-tagged GPR107 and His-tagged AT1R were expressed in HPC. Co-IP and immunoblotting analysis were performed using antibodies against His, Flag, AT1R, or GPR107. A specific interaction was readily detected between proteins (Fig. 3b). Furthermore, CO-IP followed by immunoblotting using an anti-AT1R antibody demonstrated an endogenous interaction between AT1R and GPR107 (Fig. 3c). When both the Flag-tagged GPR107 and HA-tagged clathrin were expressed in HPC, we observed an interaction between GPR107 and clathrin (Fig. 3d). After conducting transient co-expression of the Flag-tagged GPR107 and His-tagged AT1R in HPC, we observed notable co-localization between GPR107 and AT1R using confocal microscopy (Fig. 3e, f). Similarly, clathrin tagged with HA also showed significant co-localization with GPR107 in the same experiments (Fig. 3g, h). These findings indicate that GPR107 and AT1R are associated with clathrin-mediated endocytosis.

**Fig. 4:**
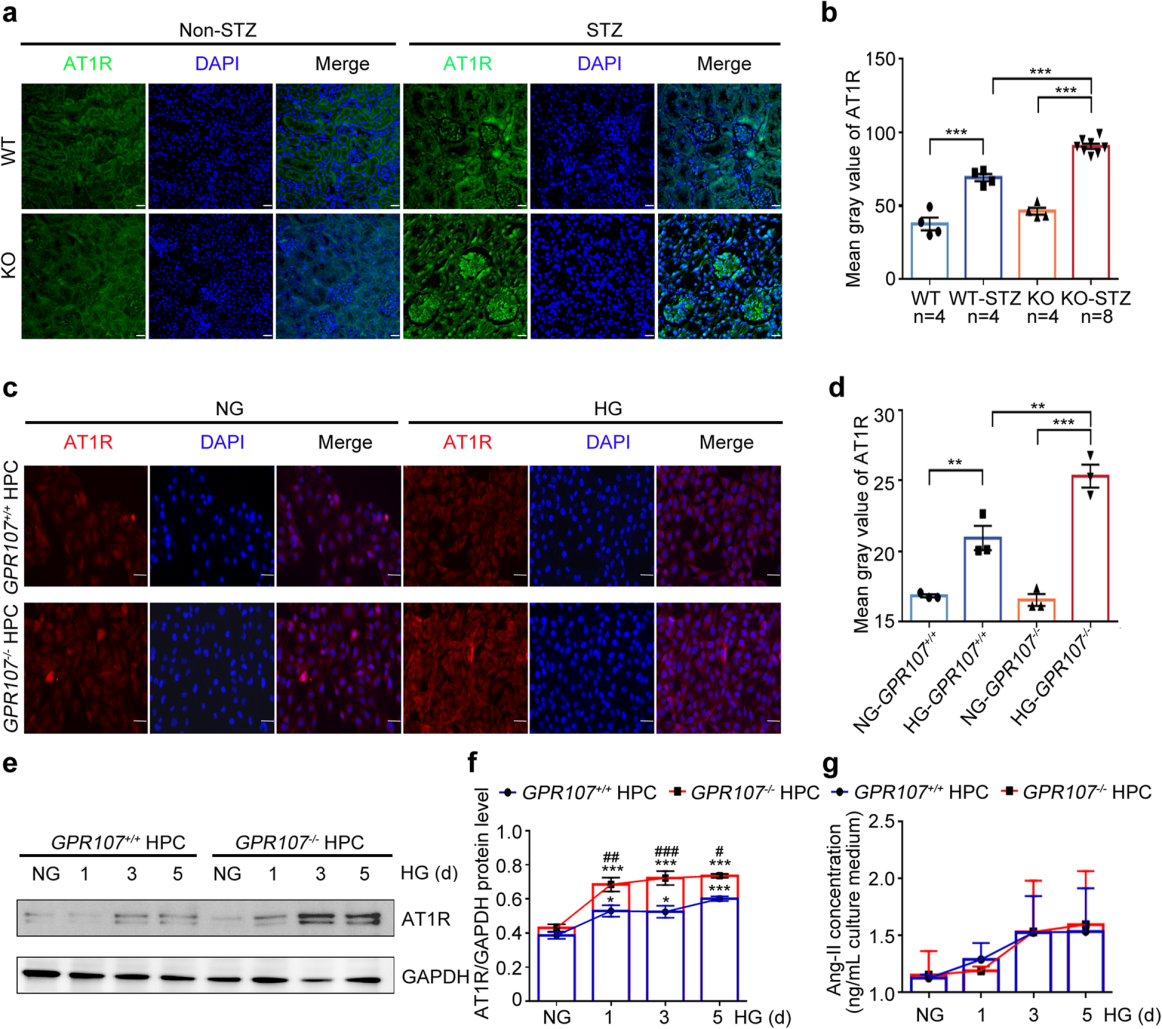
GPR107 deficiency enhances AT1R expression and Ang-II secretion in podocytes. **a**, **b** Immunostaining analysis of AT1 expression in renal tissues from the WT, KO, WT-STZ, and KO-STZ mice. *** *P* < 0.001. Original magnification × 400, bar = 20 μm. Each dot represents data obtained from 1 mouse specimen. **c**, **d** Immunofluorescence analysis for AT1R expression in HPC. ** *P* < 0.01, *** *P* < 0.001. Original magnification × 200, bar = 20 μm. **e**, **f** Western blotting analysis of AT1R expression in HPC. Representative blots of three independent experiments are shown. * *P* < 0.05, *** *P* < 0.001 compared with the *GPR107*^+*/*+^ and *GPR107*^*−/−*^ HPC under NG conditions. ^#^ *P* < 0.05, ^##^ *P* < 0.01, ^###^ *P* < 0.001 compared with the *GPR107*.^+*/*+^ HPC under HG conditions. **g** The level of Ang-II in the supernatants of HPC. Results are expressed as the mean ± SE for each group. AT1R, Angiotensin II type1 receptor. HG, high glucose (20 mM). NG, normal glucose (11.1 mM)

Since Ang-II binding triggers endocytosis of AT1R in podocytes of individuals with DN [26, 27], we expressed GFP-tagged AT1R in HPC treated with Ang-II to examine the role of GPR107 in AT1R endocytosis in podocytes. The intensity of membrane-associated GFP fluorescence of *GPR107*^+/+^ HPC cells were observed to be weaker compared to that of *GPR107*^−/−^ HPC. The fluorescence in *GPR107*^+/+^ HPC gradually decreased after treatment with Ang-II for a duration of 1 to 10 min, whereas a stable fluorescent intensity was observed in *GPR107*^−/−^ HPC treated with Ang-II. The findings indicate that deficiency of GPR107 reduces Ang-II-dependent internalization of AT1R in podocytes, leading to an increased expression of AT1R on podocyte membranes (Fig. 3i, j). Consistent with this, the glomeruli of KO-STZ mice exhibited significantly higher expression of AT1R compared with the glomeruli of WT-STZ mice. Increased AT1R was also observed in both *GPR107*^+/+^ and *GPR107*^−/−^ HPC cultured in HG (Fig. 4a-f). Upregulation of AT1R is accompanied by a substantial increase in Ang-II in the supernatants of HPC (Fig. 4g). Furthermore, when GFP-tagged AT1R is expressed in HPC treated with Ang-II, it was found that the endocytosis reduction of AT1R in *GPR107*^*−/−*^ HPC stimulated by Ang-II was restored in RC HPC (Fig. S4c, 4d). In summary, deficiency of GPR107 impairs the process of endocytosis in podocytes, which prevents the endocytosis of AT1R induced by Ang-II under high glucose conditions, thereby promoting the accumulation of AT1R in the podocytes membrane.

### The accumulation of AT1R contributes to Ca^2+^ influx in podocytes

**Fig. 5:**
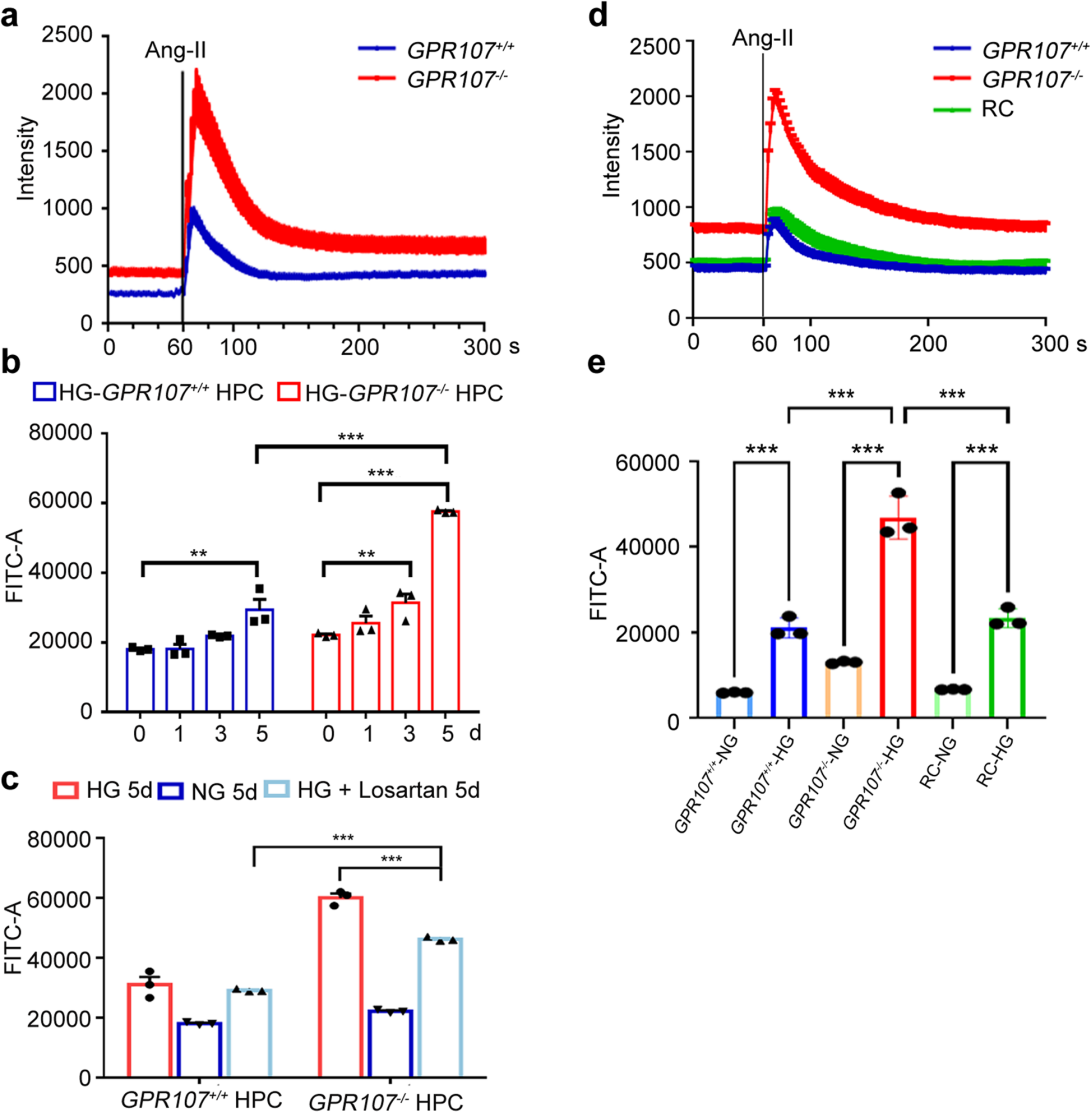
Deficiency of GPR107 results in an influx of calcium into podocytes under high glucose conditions depending on AT1R. **a** Representative images of calcium fluxes in HPC induced by Ang-II. **b**, **c** The fluorescence intensity analysis of HPC under HG conditions with or without the antagonist of AT1R (Losartan, 10 μM). ** *P* < 0.01, *** *P* < 0.001. **d** Representative images of Ca^2+^ influxes in HPC induced by Ang-II. The cells, including the *GPR107*^+*/*+^*, GPR107*^*−/−*^ and RC HPC cells. **e** The fluorescence intensity analysis of HPC under HG conditions. RC, rescue of *GPR107*.^−/−^ HPC. Results are expressed as the mean ± SE. ** *P* < 0.01, *** *P* < 0.001. HPC, human podocytes. Ang-II, Angiotensin II. AT1R, Angiotensin II type1 receptor. HG, high glucose (20 mM). NG, normal glucose (11.1 mM)

The endocytosis of AT1R by podocytes plays an important role in maintaining normal renal function because the binding of AT1R and Ang-II will activate the RAAS, leading to the activation of downstream signaling pathways and causing a large amount of Ca^2+^ influx. The increased Ca^2+^ will damage the ability of podocytes to synthesize and degrade ECM, causing GBM thickening. To verify whether GPR107 affects Ca^2+^ influx through AT1R, Fluo-4 AM labeled cells were examined using calcium ion imaging systems and flow cytometry. We found an increased Ca^2+^ influx triggered by Ang-II in *GPR107*^−/−^ HPC compared to *GPR107*^+/+^ HPC when exposed to HG (Fig. 5a). However, overexpression of *GPR107* had no impact on Ca^2+^ influx in HPC when stimulated with Ang-II. Meanwhile, no enhancement of COL4 expression was observed when HG was stimulated in OE HPC (HPC overexpression of *GPR107*) (Fig. S5a-5c). These results suggest that enhancing the expression of GPR107 in HPC can inhibit the increase of COL4 expression induced by HG stimulation. Given the association between increased levels of Ang-II and high glucose levels in patients with DN, we sought to investigate the role of GPR107 in regulating Ca^2+^ influx alterations through AT1R in HPC exposed to HG. HPC cells were incubated in HG for 1 to 5 days. A noticeable increase in Ca^2+^ concentrations was observed in both *GPR107*^+/+^ and *GPR107*^−/−^ HPC after a 5-day exposure to HG, compared to NG (Fig. 5b, Fig. S5d). After 5 days of culture in HG, *GPR107*^−/−^ HPC exhibited a significantly increased in Ca^2+^ concentration, compared to that in *GPR107*^+/+^ HPC. Additionally, after a 5-day culture in HG, a significant reduction in Ca^2+^ influx was observed in the *GPR107*^−/−^ HPC treated with Losartan (an AT1R antagonist), as compared to the control group. Losartan, an angiotensin receptor blockers, plays a protective role in diabetic nephropathy and hypertension [28, 29]. However, no substantial alterations in Ca^2+^ influx were observed in *GPR107*^+/+^ HPC (Fig. 5c, Fig. S5e). The Ang-II-induced increase in Ca^2+^ inflow in *GPR107*^*−/−*^ HPC was not seen in RC HPC (rescue of *GPR107*-deficient HPC), the changes are similar to *GPR107*^+*/*+^ HPC (Fig. 5d). In addition, flow cytometry showed that compared with *GPR107*^*−/−*^ HPC, RC HPC could significantly reduce the increase of Ca^2+^ concentration induced by HG (Fig. 5e). These findings suggest that deficiency of GPR107 leads to a decrease in AT1R endocytosis in podocytes, which in turn enhances Ang-II–mediated activation and subsequently increases Ca^2+^ influx.

### Deficiency of GPR107 promotes the synthesis of COL4 in podocytes and inhibits its degradation under high glucose conditions

**Fig. 6:**
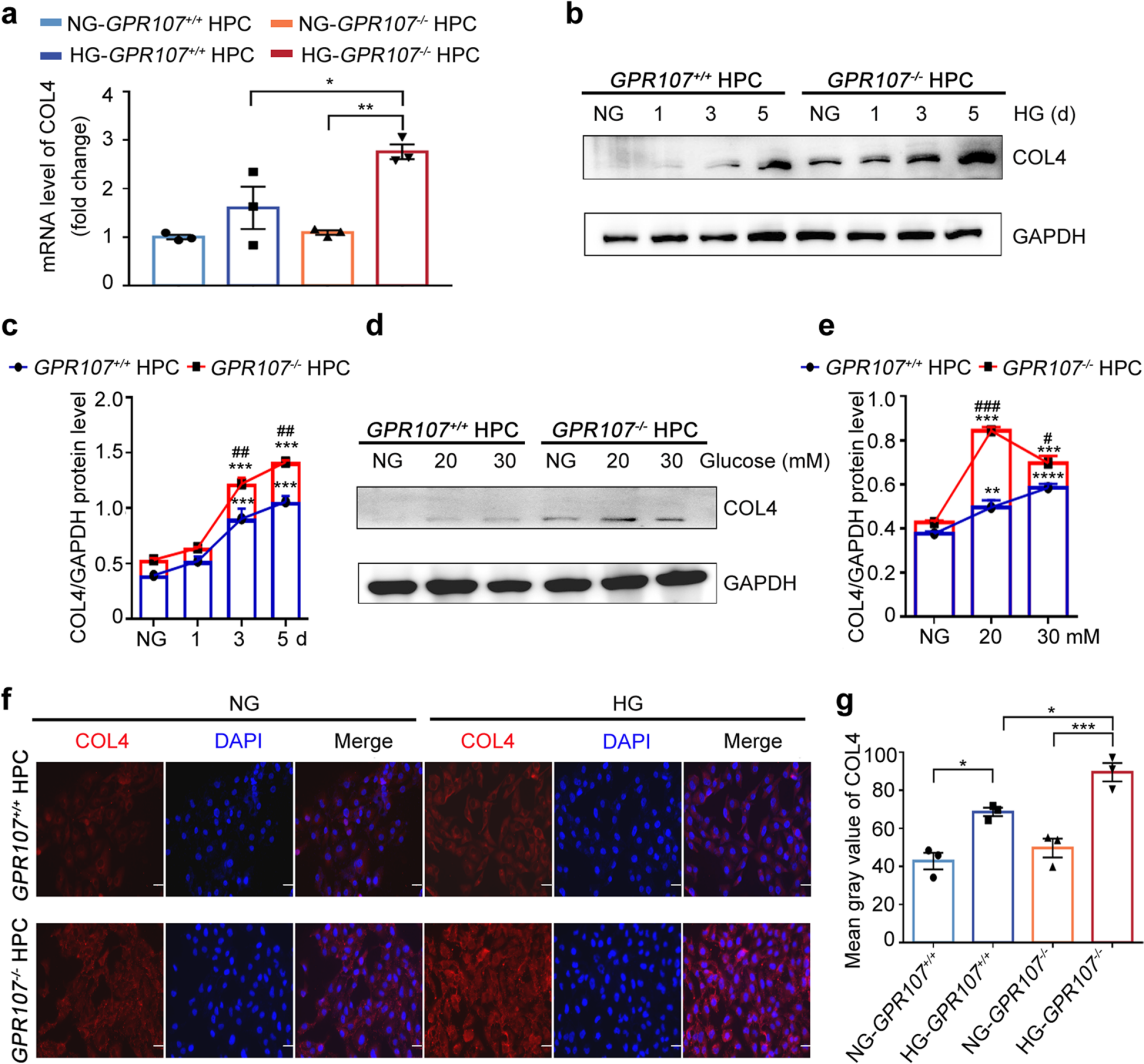
GPR107 deficiency promotes an increase of COL4 synthesis in podocytes under the HG conditions. **a** RT-qPCR analysis of *COL4* expression in HPC. * *P* < 0.05. **b**, **c** Western blotting analysis of COL4 expression in HPC cultured in HG for 1, 3, and 5 days. * *P* < 0.05, ** *P* < 0.01, *** *P* < 0.001 compared with the *GPR107*^+*/*+^ HPC cultured in NG. ^#^ *P* < 0.05, ^##^ *P* < 0.01, ^###^ *P* < 0.001 compared with the *GPR107*^+*/*+^ HPC grown in HG. **d**, **e** Western blotting analysis of COL4 expression in HPC cultured in different concentration of glucose for 5 days. * *P* < 0.05, ** *P* < 0.01, *** *P* < 0.001, **** *P* < 0.0001 compared with the *GPR107*^+*/*+^ HPC cultured in NG. ^#^ *P* < 0.05, ^##^ *P* < 0.01, ^###^ *P* < 0.001 compared with the *GPR107*.^+*/*+^ HPC grown in the medium with glucose at concentration of 20 mM or 30 mM, respectively. **f**, **g** Immunofluorescence analysis for COL4 expression in podocytes cultured in medium with either NG or HG for 5 days. * *P* < 0.05, *** *P* < 0.001. Results are expressed as the mean ± SE for three samples from each group. Representative blots from three independent experiments are presented for quantitative analysis. Original magnification × 200, bar = 20 μm. HG, high glucose (20 mM). NG, normal glucose (11.1 mM)

GBM thickening can be affected by the influx of Ca^2+^ and the increase of Ca^2+^ concentration in podocytes caused by HG. The IHC results in Fig. 1q showed the accumulation of COL4 in the thickened GBM. An increase in GBM thickening in DN may be linked to the synthesis of COL4 in podocytes. To examine whether GPR107 deficiency affects COL4 expression in podocytes stimulated by high glucose, we conducted the following experiments. Real time fluorescence quantitative (RT-qPCR) analysis demonstrated that the 5-days HG culturing led to elevated expression of *COL4* in both *GPR107*^−/−^ and *GPR107*^+/+^ HPC, in comparison to NG (Fig. 6a). Western blotting analysis demonstrated that HG induced expression of COL4 in a dose-dependent and time-dependent manner, compared to *GPR107*^+/+^ HPC, *GPR107*^−/−^ HPC exhibited a more pronounced dose-dependent and time-dependent responses to HG (Fig. 6b-e). Immunofluorescence analysis also demonstrate a significant increase in the levels of COL4 in *GPR107*^−/−^ HPC in HG compared to *GPR107*^+/+^ HPC (Fig. 6f, g). These findings indicate that deficiency of GPR107 enhances synthesis of COL4 in podocytes subjected to HG.

**Fig. 7:**
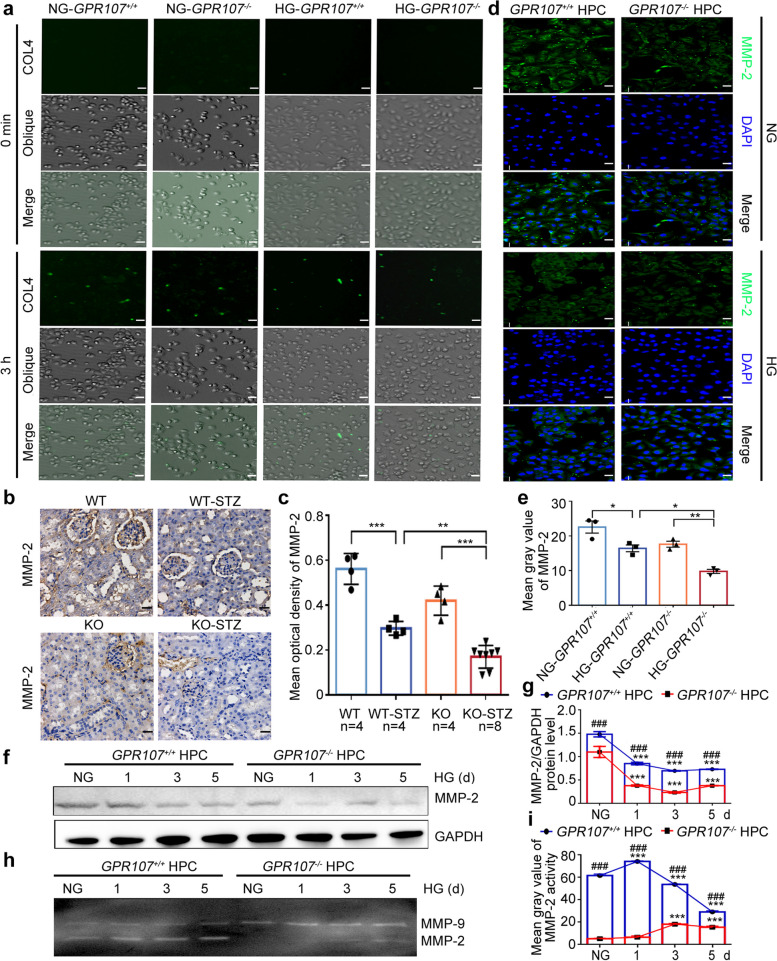
GPR107 deficiency leads to limited expression and activity of MMP-2 in the ECM of podocytes under the HG. **a** Green fluorescent degradation products of DQ-COL4 in the ECM of HPC. Original magnification × 100, bar = 20 μm. **b**, **c** Immunostaining analysis for MMP-2 expression in renal tissues from the WT, KO, WT-STZ, and KO-STZ mice. ** *P* < 0.01, *** *P* < 0.001. Original magnification × 400, bar = 20 μm. Each dot represents data obtained from 1 mouse specimen. **d**, **e** Immunofluorescence analysis for MMP-2 expression in HPC cultured in NG or HG medium. * *P* < 0.05, ** *P* < 0.01. Original magnification × 200, bar = 20 μm. **f**, **g** Western blots analysis of MMP-2 expression in HPC incubated in medium with HG for 0, 1, 3, and 5 days. *** *P* < 0.001 compared to the *GPR107*^+*/*+^ and *GPR107*^*−/−*^ HPC cultured in NG. ^###^ *P* < 0.001 compared with the *GPR107*^+*/*+^ HPC cultured in HG medium. **h**, **i** Gelatin enzyme assay for the activities of MMP-2 and MMP-9 in the supernatants of *GPR107*^+*/*+^ and *GPR107*^*−/−*^ HPC incubated in medium with HG for 0, 1, 3, 5 days. *** *P* < 0.001 compared to the *GPR107*^+*/*+^ and *GPR107*^*−/−*^ HPC incubated in the medium with NG. ^###^ *P* < 0.001 compared with the *GPR107*.^+*/*+^ HPC grown in HG medium. Data are shown as the mean ± SE for three samples in each group. Representative blots from three independent experiments are presented for quantitative analysis. KO, knockout. WT, wild type. STZ, streptozotocin. HG, high glucose (20 mM). NG, normal glucose (11.1 mM)

Additionally, due to the accumulation of COL4, GBM in DN thickens, which may be associated with a decrease in the degradation of COL4 in podocyte ECM. To examine this possibility, we cultured HPC with the DQ-COL4 for 0–3 h in NG or HG. After co-culturing for 0.5 h, we observed the extracellular fluorescent degradation products progressively intensified over time in all HPC (Fig. 7a and Fig. S6a). Both *GPR107*^+/+^ and *GPR107*^−/−^ HPC displayed weaker signals in HG, compared to NG. Furthermore, *GPR107*^−/−^ HPC in HG manifested a comparatively diminished signal in contrast to *GPR107*^+/+^ HPC. These results indicate that HG inhibits the degradation of COL4 in the ECM of podocytes, especially in *GPR107*^−/−^ podocytes.

MMP-2 and MMP-9 contribute to the degradation of COL4 in the ECM of podocytes. RT-qPCR was used to detect which plays an important role in it. The results showed that *MMP-9* exhibited consistent expression levels in both the *GPR107*^+/+^ and *GPR107*^−/−^ HPC in HG. However, *MMP-2* displayed a significant decrease in the *GPR107*^−/−^ HPC in HG compared to NG (Fig. S6b). Furthermore, renal tissues from the STZ-induced DN exhibited a significant decrease in the levels of MMP-2 when compared to normal tissues. Specifically, the KO-STZ mice exhibited lower MMP-2 levels in comparison to the WT-STZ mice (Fig. 7b, c). When HPC cells were cultured in HG for 0 to 5 days, the *GPR107*^+/+^ HPC exhibited expression of MMP-2, whereas the *GPR107*^*−*/−^ HPC exhibited reduced levels of MMP-2 (Fig. 7d-g).

The activities of matrix metalloproteinases in the medium of HPC cultured in HG were determined by using a gelatin enzyme assay. Representative results of zymography data are shown in Fig. 7h-i. There were no significant differences in the enzymatic activities of MMP-9 between the *GPR107*^+/+^ and *GPR107*^−/−^ HPC. The activities of MMP-2 were easily detected in the *GPR107*^+/+^ HPC cultured in HG. However, weakly detectable activities of MMP-2 were observed in the medium of *GPR107*^−/−^ HPC. Therefore, the above results prove that GPR107 deficiency leads to limited expression and activity of MMP-2, resulting in decreased COL4 degradation in the ECM of podocytes in HG.

### Abnormal expression of COL4 and matrix metalloproteinase 2 (MMP-2) in GPR107-deficient podocytes is associated with the activation of the AT1R/Ca^2+^ signaling pathway

**Fig. 8:**
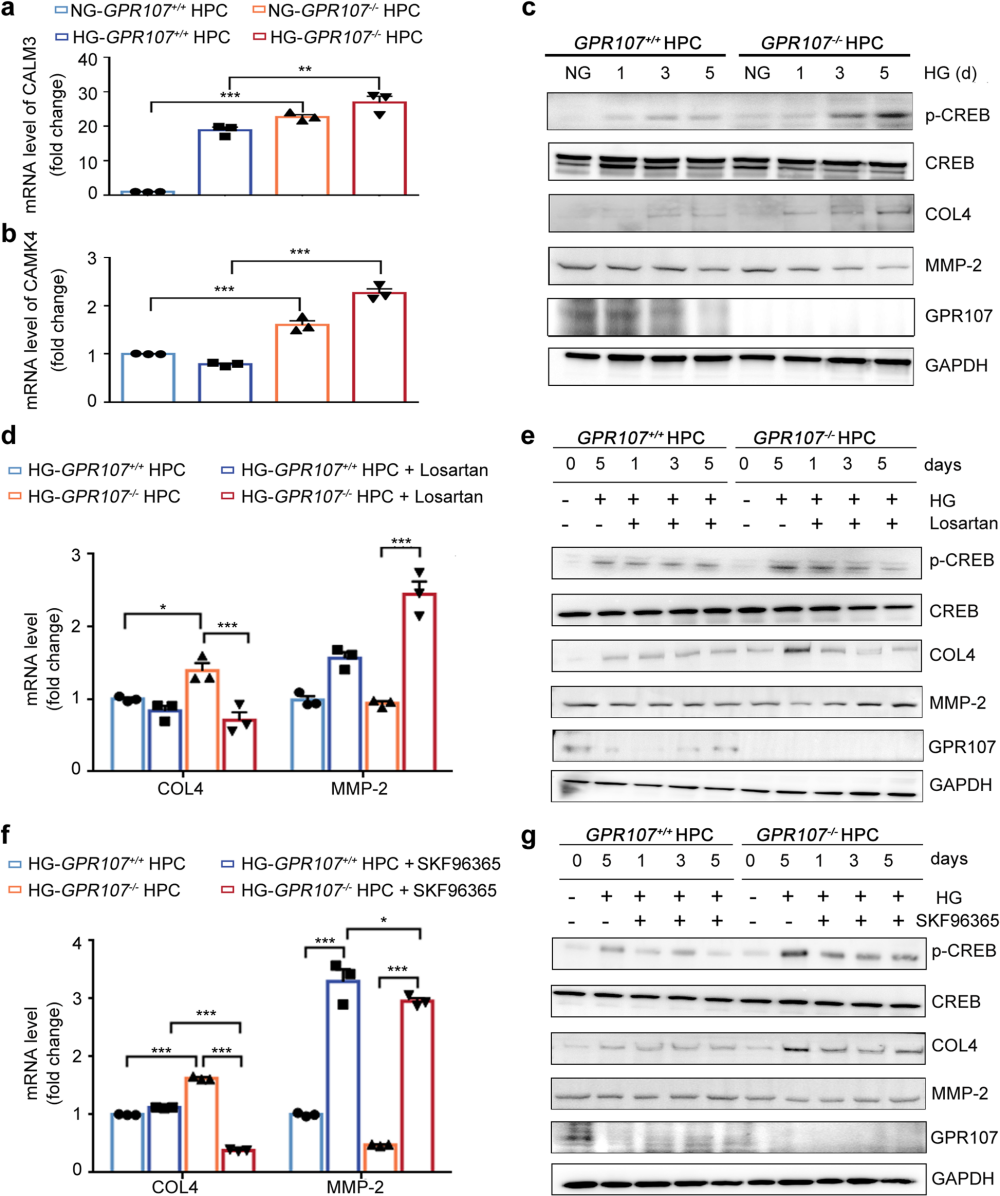
Regulating the expression of COL4 and MMP-2 mainly depends on activation of the AT1R/Ca^2+^/p-CREB signal pathway in the *GPR107* deficient podocytes. **a**, **b** RT-qPCR was used to detect the expression levels of crucial signal molecules related to the Ca^2+^ pathway. ** *P* < 0.01, *** *P* < 0.001. **c** Western blotting analysis for the expressions of COL4, MMP-2, GPR107 and CREB, as well as the activation of CREB (phosphorylated-CREB, p-CREB). **d** The mRNA levels of *COL4* and *MMP-2* were detected using the RT-qPCR analysis. Both the *GPR107*^+*/*+^ and *GPR107*^*−/−*^ HPC were cultured in HG medium, with or without the addition of Losartan, for a duration of 5 days. * *P* < 0.05, ** *P* < 0.01. **e** Losartan exhibits a reversing effect on the expression patterns of COL4, MMP-2 and GPR107, as well as levels of the p-CREB in HPC. **f** RT-qPCR was used to detect the levels of expression of *COL4* and *MMP-2* in HPC with or without pretreatment with the SKF96365 (a blocker of the Ca.^2+^ channels) for 30 min. **g** The SKF96365 displays a reversing effect on the expression patterns of COL4, MMP-2 and GPR107, as well as levels of the *p*-CREB in HPC. Results are expressed as the mean ± SE for three samples from each group. HPC, human podocytes. HG, high glucose (20 mM). NG, normal glucose (11.1 mM). Calmodulin 3 (CALM3), Calcium/calmodulin-dependent protein kinase IV (CAMK4)

Our hypothesis suggests that activation of the AT1R/Ca^2+^ signal may be responsible for the abnormal expression of COL4 and MMP-2 in the GPR107 deficient podocytes. The classical Ca^2+^ signaling pathway involves several key signal molecules, namely Ca^2+^, calmodulin (CALM), calmodulin-dependent protein kinases (CaMKs), and Camp response element-binding protein (CREB) [30–32]. In order to verify our hypothesis, we conducted validation from mRNA and protein levels. We observed a significant increase in the mRNA level of *Calmodulin 3* (*CALM3*) and *calcium/calmodulin-dependent protein kinase IV* (*CAMK4*) in *GPR107*^−/−^ HPC in HG, compared to *GPR107*^+/+^ HPC (Fig. 8a, b). Examination of the levels of p-CREB, COL4, MMP-2 and GPR107 in HPC in HG showed a noticeable increase in the expression of COL4 in *GPR107*^−/−^ HPC cultured in HG for 0, 1, 3 and 5 days. This increase was accompanied by an elevated expression of p-CREB. However, the expression of MMP-2 and GPR107 decreased gradually (Fig. 8c). To investigate further, we treated both the *GPR107*^+/+^ and *GPR107*^−/−^ HPC using SKF-96365 (a blocker of store-operated Ca^2+^ entry) or Losartan. SKF-96365, an inhibitor of transient receptor potential canonical (TRPC) channels, attenuates Ang-II-induced elevations in cytosolic calcium concentration [33, 34]. After 5 days of treatment with SKF-96365 (or Losartan), both *GPR107*^+/+^ and *GPR107*^−/−^ HPC exhibited a notable reduction in *COL4* expression at mRNA levels (Fig. 8d, f). In contrast, SKF-96365 (or Losartan) treatments led to an increased mRNA expression of *MMP-2* in both *GPR107*^+/+^ and *GPR107*^−/−^ HPC in HG. Further, immunoblotting analysis showed Losartan and SKF-96365 reversed the HG-induced changes in COL4 and MMP-2 expression in *GPR107*^−/−^ and *GPR107*^+/+^ HPC (Fig. 8e, g). The elevated p-CREB, which was induced by HG for a period of 5 days, exhibited a gradual reduction in the *GPR107*^−/−^ HPC treated with either Losartan or SKF-96365 for 1, 3, and 5 days. These results support the view that HG promotes activation of the AT1R/Ca^2+^/p-CREB signal pathway to regulate expression of COL4 and MMP-2 in the *GPR107*-deficient HPC, inhibition of AT1R or Ca^2+^ signaling pathway can weaken this effect.

## Discussion

The thickening of GBM, as a characteristic pathological alteration in DN, is primarily attributed to the accumulation of ECM components within the GBM. Since COL4 serves as a key component of the ECM and provides structural support to the GBM, the abnormal accumulation of COL4 in the ECM is considered the primary cause of GBM thickening in DN [11, 12, 35]. Increased production by mesangial cells (endothelial cells) and podocytes, as well as dysregulated degradation [35–37], contribute to the deposition and accumulation of COL4 in the ECM of the GBM. Additionally, the process of podocytes endocytosis plays a crucial role in regulating the composition of the glomerular capillary wall to ensure efficient filtration [14, 38, 39]. However, little is known about the endocytosis of COL4 by podocytes in DN.

GPR107 is a member of the GPCR superfamily [23]. We have previously observed that the deletion of *Gpr107* leads to an embryonic lethal phenotype, which is characterized by a reduction in expression of the cubilin-megalin endocytic receptor [25]. Functional analysis has shown that fibroblasts lacking *Gpr107* exhibit reduced internalization of transferrin, decreased uptake of cargo through the low-density lipoprotein receptor-related protein-1, and inhibition of CME. Cubilin and megalin are predominantly expressed in epithelial cells, particularly in podocytes [40, 41], indicating a potential role of GPR107 in regulating podocytes functions, such as endocytosis, specifically in DN. In this study, expression of GPR107 considerably decreased in the renal tissues of patients with DN and mice with STZ-induced DN. The expression level of GPR107 in clinical kidney specimens was negatively correlated with the degree of kidney damage. The results of animal experiments are consistent, the deficiency of GPR107 is closely related to GBM thickening. The increased thickness of the GBM primarily arises from the accumulation of COL4 in both *Gpr107* (KO)-STZ and WT-STZ mice. Furthermore, the KO-STZ mice exhibited a greater accumulation of COL4 in comparison to the WT-STZ mice. This finding is consistent with the more pronounced degree of kidney damage observed in *Gpr107*-(KO) mice with DN.

A COL4 Internalization assay confirmed that podocytes are capable of internalizing COL4 from the ECM within a short period of time (within 30 min). However, the endocytosis process is significantly inhibited when exposed to high glucose conditions. GPR107 deficiency effectively inhibits the process of COL4 internalization in podocytes under both normal and high glucose conditions. Nevertheless, this change disappeared after the rescue of *GPR107*^*−/−*^ HPC. Hence, the decreased COL4 internalization by podocytes presents itself as a conceivable factor contributing to increased COL4 deposition within the ECM of podocytes.

Previous studies have demonstrated that hyperglycemia induces an alteration in the pattern of podocytes integrin expression, resulting in an enhanced synthesis of COL4 and a reduced expression of matrix metalloproteinases (specifically MMP-2) [12, 36, 42–44]. The present study has demonstrated, at both the mRNA and protein levels, that high glucose induces enhanced *COL4* synthesis and suppresses *MMP-2* synthesis in podocytes. Moreover, these high-glucose effects are more pronounced when *GPR107* is deficient. MMP-2 activities in the podocytes ECM are only weakly detectable, possibly due to a decrease in MMP-2 expression and secretion in *GPR107*-deficient podocytes when exposed to high glucose. Hence, an imbalance between the production and degradation of COL4 also contributes to the accumulation of COL4 in the ECM of podocytes in DN.

In a hyperglycemic medium, the ECM of podocytes exhibits elevated levels of Ang-II, leading to the internalization of AT1R through CME [45, 46]. Additionally, Ang-II can bind to AT1R and facilitate extracellular signal transduction [21, 47, 48]. A recent study has demonstrated that the inhibition of endocytosis of clathrin-mediated AT1R in podocytes exacerbates glomerular injury [18]. Ang-II-induced endocytosis of AT1R in podocytes may be crucial for maintaining the balance of AT1R signaling and may contribute to the normal functions of the kidney. Both AT1R and GPR107 interact with clathrin in podocytes. Under high glucose conditions, it was observed that AT1R internalization in *GPR107*^*−/−*^ HPC decreased leading to increased Ca^2+^ concentration, and increased reactivity to Ang-II induced Ca^2+^ flow. Rescue of *GPR107*^*−/−*^ HPC restored these changes are gone. Given the high expression of CaMK and elevated Ca^2+^ levels in *GPR107*-deleted podocytes stimulated by high glucose, GPR107 deficiency appears to be beneficial for the activation of the AT1R/Ca^2+^ signaling pathway. Ang-II could also induce the phosphorylation of CaMK II and CREB in cultured podocytes [32]. Consequently, the activation of the AT1R/Ca^2+^ pathway leads to up-regulation of COL4 expression and down-regulation of MMP-2 expression through p-CREB.

The present study attempted to explore the mechanisms by which the deficiency of GPR107 affects the expression of COL4 and MMP-2 in podocytes under high glucose conditions. In the current study, we examined a limited number of clinical specimens from individuals with T1DM, as well as mice with STZ-induced persistent hyperglycemia on a congenic C57BL/6 background, to mimic T1DM. To further investigate the functions of GPR107 in DN, it will be necessary to expand the number of clinical samples of both T1DM and T2DM. Additionally, future studies should involve backcrossing the genetic mouse models onto a susceptible DN background. Another potential limitation of this investigation is the focus on GPR107 functions in podocytes. Even though our data provide strong evidence for a crucial role of GPR107 in podocytes, we cannot exclude the possibility that other cells in the kidney (e.g., epithelial cells, endothelial cells, and glomerular mesangial cells) may contribute to kidney damage. This is because GPR107 is expressed primarily in both the renal medulla and renal cortex.

Considering the results presented here, our study provides new and original insights into the functions of GPR107 in the accumulation of COL4, leading to thickening of the GBM in DN. Absence of GPR107 in DN can aggravate kidney injury. The expression of GPR107 is negatively correlated with renal function, and the absence of GPR107 aggravates renal injury in DN. GPR107 expression is a hallmark of DN severity. We propose that increased GPR107 activity may inhibit the progression of DN. While neuronostatin, a proposed GPR107 activator [49], shows potential for regulating GPR107, its hyperglycemic effect limits its use in DN [50–52]. Developing novel small-molecule GPR107-targeting compounds may help regulate the balance of COL4 in the ECM of podocytes within the GBM in DN patients.

## Conclusion

The expression of GPR107 in renal tissues of DN patients and model mice is significantly decreased. The absence of GPR107 promoted the accumulation of COL4 in ECM and aggravated GBM thickening, renal function injury and DN progression. The deficiency of GPR107 inhibits endocytosis of COL4 and AT1R in podocytes and increases the expression of AT1R on the cell membrane, resulting in increased Ca^2+^ influx and activation of AT1R/Ca^2+^/p-CREB signaling pathway. Activation of the signaling pathway increased the synthesis of COL4 in podocytes, at the same time, decreased synthesis of MMP-2 and accompanied by it’s decreased activity, which weakened the degradation of COL4. In summary, GPR107 prevents COL4 accumulation in ECM, and reduced GPR107 expression correlates with DN severity. Further research is needed to determine whether targeting GPR107 with small molecules represents a viable therapeutic strategy to regulate COL4 levels in the GBM of DN patients.

## Materials and methods

### Reagents

The antibodies used in the current study were listed in the Table S1. Both STZ and Tamoxifen were purchased from Sigma-Aldrich. The targeting vectors (TV1 and TV2), as well as the expression vectors, including a Flag-tagged GPR107 (pCMV-Tag2B-GPR107), a His-tagged AT1R (pcDNA3.1-His-C-AT1R), a HA-tagged Clathrin (pcDNA3.1-HA-N-Clathrin), along with a GFP-tagged AT1R (EGFP-AT1R), were constructed in our laboratory. Dye-quenched Collagen IV (DQ-COL4), Mouse PCR Kit, TRIzol™ and Lipofectamine 3000 reagent were purchased from Invitrogen. Masson’s trichrome staining kits and PAS staining kits were purchased from Solarbio. Gelatin-Zymography Kit and Fluo-4 AM was purchased from Xinfan Biotechnology and Beyotime. Evo M-MLV RT Premix and SYBR Green Premix Pro Taq HS qPCR Kits were purchased from Accurate Biology. The AT1R antagonist, Losartan, was purchased from MCE. The calcium channel blocker, SKF-96365, was obtained from Abcam.

### Patients and samples

Normal renal tissues were obtained from 7 individual patients with nephrectomy specimens for the treatment of urinary system tumors. The opposite of the tumor was pathologically diagnosed as normal renal tissue without tumor invasion, which was used as healthy control. Clinical samples were collected from 12 individual patients with DN.

### Diabetes model

All mice in this study were of a congenic C57BL/6 background. Details of the generation and identification of the *Gpr107* knockout mice (KO) are described in the Supplementary Methods. To establish animal models of DN, Eight-week-old mice were intraperitoneally injected with a single dose of STZ at the concentration of 50 mg/kg for 5 consecutive days. Age-matched control mice received 0.1 mol/L sterile sodium citrate for 5 consecutive days. Mice were randomly divided into four groups: WT mice (*n* = 4), KO mice (*n* = 4), WT mice injected with STZ (WT-STZ mice, *n* = 4), and KO mice injected with STZ (KO-STZ mice, *n* = 8).

### Urine and cell culture medium examination

The urinary albumin-to-creatinine ratio (UACR) and 24 h urine protein were assessed by a biochemical instrument (Roche). In order to determine the levels of Ang-II produced by HPC, the supernatants of cells collected over a 24-h period were concentrated tenfold. The concentration of Ang-II was measured using the chemiluminescent analyzer (Autobio).

### Immunohistochemistry and immunofluorescence

Immunohistochemistry and immunofluorescence analysis was performed using conventional standard techniques as described elsewhere [53]. Cell or kidney specimens were stained with specific antibodies in Supplementary Table S1. The images were captured using the TissueFAXS Spectra imaging system (TissueGenostics). Double immunofluorescence was photographed using confocal microscopy (Zeiss) to observe the co-localization. The Image J software was used for image analysis.

### Histologic analysis

The histological samples of the kidneys were fixed in 4% polyformaldehyde for 48 h. The 4 µm sections were obtained from kidney tissues embedded in paraffin. The sections were stained with hematoxylin and eosin (H&E)、Masson’s trichrome, and Periodic Acid Schiff (PAS) for histological analysis. The collagen volume fraction and glomerular area were assessed as previously described [54, 55]. Briefly, assessment of the collagen volume fraction was performed in a blinded manner using at least 10 random fields from each kidney slice. This assessment was based on sections stained with Masson’s trichrome and was facilitated by the software Image J. Glomerular area was determined by tracing the outline of the Bowman’s capsule using PAS staining and the software Image J. A minimum of 50 randomly selected superficial glomeruli per mouse were chosen for analysis. Images were captured with TissueFAXS Spectra.

### Transmission electron microscopy

The processing of samples was carried out in the Electron Microscope Center of Anhui Medical University as previously mentioned [56]. The images were captured, observed, and the thickness of GBM was measured using a transmission electron microscope (Thermo Scientific).

### Cell culture and treatment

The human podocytes cell line, HPC, were cultured in RPMI 1640 medium with 10% fetal bovine serum (FBS). TECs were extracted according to the method described previously [57]. HK-2, HKC and TECs were cultured in DMEM/F12 medium with 10% FBS. MCs were cultured in DMEM medium with 10% FBS, The *GPR107*^−/−^ HPC clone was generated by utilizing the CRISPR-Cas9 technique and identified by PCR analysis using the specific primers: forward: 5’ -CAGAGGAGACCACGTTAGAAGT-3’, and reverse: 5’—CTGGGCTCAGGT GAAGAGATG-3’. An *GPR107*-overexpressing HPC cell line (OE HPC) was generated by transfecting HPC with a plasmid containing the *GPR107* cDNA using liposome-mediated transfection. *GPR107*-deficient HPC (*GPR107*^*−/−*^ HPC) were rescued (RC HPC) by transfection with a pCMV-Tag2B plasmid containing the GPR107 cDNA, followed by G418 selection. Cell transfection was performed with the Lipofectamine 3000 according to the manufacturer’s instructions. Cells were treated with the medium containing either normal glucose (11.1 mmol/L) ± 9 mM mannitol (NG) or high glucose (20 mmol/L) (HG) for the indicated time intervals. Losartan (10 μM) or SKF-96365 (40 μM) was added to inhibit AT1R or calcium channels in HPC once the cells reached 80% confluence.

### Real-time quantitative polymerase chain reaction analysis

Details are provided in the Supplementary Methods.

### Western blots

Details are provided in the Supplementary Methods.

### RNA Sequencing

Details are provided in the Supplementary Methods.

### Gelatin zymography

Gelatin zymography was performed according to the methods described in a previous publication [58]. Briefly, Conditioned media samples were equally loaded and applied without being heated or reduced to a 10% SDS–polyacrylamide gel containing 1 mg/ml of gelatin. After electrophoresis, the gel was washed twice with distilled water, and incubated for 48 h with 1 × buffer A at room temperature. Then, buffer A was replaced with 1 × buffer B and incubated overnight at room temperature. The gel was stained with a solution of Coomassie Brilliant Blue for 2 h and subsequently de-stained using a solution composed of 50% methanol and 10% acetic acid. Clear bands represent areas of proteolytic activity. Images were quantified using the image J software.

### Endocytosis assay

COL4 internalization was performed according to the protocols described in a previous article [18, 59]. Briefly, the cells were co-cultured with the quenched fluorescent substrate DQ-COL4 (25 μg/ml) at 37 ℃ for different time durations under normal or high glucose conditions, then rapidly rinsing the cells with ice-cold PBS for 3 times and fixed in 4% paraformaldehyde. Images were captured under the reverse fluorescence microscope. To assess the presence of internalized AT1R on the cellular membrane, we transiently transfected HPC with the GFP-tagged AT1R vector (EGFP-AT1R) using Lipofectamine 3000. The transfected cells were then seeded in 12-well plates and stimulated with Ang-II (100 μM) for different time durations at 37 ℃. Images were obtained by Celldiscoverer 7 (Zeiss). A quantitative analysis of the fluorescence intensity was performed using the Image J software.

### Co-immunoprecipitation (Co-IP)

Details are provided in the Supplementary Methods.

### Live cell proteolysis assay

ECM degradation assays were performed according to the methods described in a previous publication [60]. Briefly, prior to seeding the cells, the bottoms of the cell-culture dish were coated with 25 μg/ml DQ-COL4 and incubated for 10 min at 37 °C. Subsequent steps were carried out at 37 °C. The cells were seeded onto the coated cell-culture dish and incubated in the culturing chamber of the Celldiscoverer 7 at 37℃ with 5% CO_2_. Images of the live cells were captured at 0.5, 1, 2, and 3 h. Resulting green fluorescence was observed in the extracellular matrix of HPC, indicating degradation products of COL4. A quantitative analysis of the fluorescence intensity was performed with Image J software.

### Intracellular Ca^2+^ activity, Ca^2+^ concentration and Ca^2+^ imaging

Details are provided in the Supplementary Methods.

### Statistical analysis

Data was presented as mean ± SE as appropriate. Differences between two groups were analyzed by unpaired two-tailed *t*-tests. Spearman correlation analysis was used to evaluate the correlation between the two variables. ANOVA and Tukey’s post hoc tests were applied for comparisons between more than two groups using Prism version 9.5 (GraphPad Software). *P* < 0.05 indicates statistically significant differences.

## Supplementary Information

Supplementary Material 1. Supplementary Material and Methods. Table S1. Antibodies Table (used in current study). Table S2. Probe sequences of MLPA. Table S3. Primer sequences for PCR. Table S4. Primer sequences for real-time PCR. Fig. S1. Expression of GPR107 in different renal cell lines and the generation of conditional knock out *Gpr107* mice. Fig. S2. The blood glucose levels and body weights of mice and identification of *GPR107*-deficient HPC. Fig. S3. GPR107 deletion decreases endocytosis of COL4 within podocytes and identification of rescue of *GPR107*^-/-^ HPC. Fig. S4. The endocytosis of COL4 and AT1R in podocytes, Fig. S5. The responses to Ang-II-induced Ca^2+^ influxes or high glucose-induced Ca^2+^ concentration and upregulation of COL4 in OE HPC. Fig. S6. High glucose inhibits the degradation of COL4 in the extracellular matrix of podocytes and RT-qPCR analysis for the expression levels of *MMP-2* and *MMP-9* in HPC.

## Data Availability

The RNA-seq data supporting the findings of this study are openly available in Gene Expression Omnibus (GEO) repository at GSE243552.
