# Supplementary material for: G protein-coupled receptor 107 deficiency promotes development of diabetic nephropathy
Source: Mol Biomed. 2025 Feb 11;6:10. doi: 10.1186/s43556-025-00250-1 (PMC11814420; doi:10.1186/s43556-025-00250-1)
Supplement: Supplementary file 1 — Supplementary Material 1. Supplementary Material and Methods. Table S1. Antibodies Table (used in current study). Table S2. Probe sequences of MLPA. Table S3. Primer sequences for PCR. Table S4. Primer sequences for real-time PCR. Fig. S1. Expression of GPR107 in different renal cell lines and the generation of conditional knock out Gpr107 mice. Fig. S2. The blood glucose levels and body weights of mice and identification of GPR107-deficient HPC. Fig. S3. GPR107 deletion decreases endocytosis of COL4 within podocytes and identification of rescue of GPR107-/- HPC. Fig. S4. The endocytosis of COL4 and AT1R in podocytes, Fig. S5. The responses to Ang-II-induced Ca2+ influxes or high glucose-induced Ca2+ concentration and upregulation of COL4 in OE HPC. Fig. S6. High glucose inhibits the degradation of COL4 in the extracellular matrix of podocytes and RT-qPCR analysis for the expression levels of MMP-2 and MMP-9 in HPC. [file 43556_2025_250_MOESM1_ESM.docx]

**Supplementary data**

**G protein-coupled receptor 107 Deficiency**

**Promotes Development of Diabetic Nephropathy**

Deping Xu^1，2^, Ziwen Tong^1^, Ping Yang^1^, Qiong Chen^1^, Suhua Wang^1^, Wei Zhao^1^, Linzi Han^1^, Yin Yu^3^, Ruyue Xu^1^, Min Zhang^4^, Chunlin Cai^5^, Deguang Wang^6^, Dandan Zang^7^, Guoling Zhou^8, *^, Haisheng Zhou^1,7, *^

^1^Department of Biochemistry and Molecular Biology, Anhui Medical University. Hefei, China.

^2^The Clinical Laboratory, Hefei Affiliated Hospital to Anhui Medical University, the Second People's Hospital of Hefei. Hefei, China.

^3^Department of Pathology, the First Affiliated Hospital of Anhui Medical University. Hefei, China.

^4^The Clinical Laboratory, the First Affiliated Hospital of Anhui Medical University. Hefei, China.

^5^Department of Pathophysiology, Anhui Medical University, Hefei, China.

^6^Department of Nephrology, the Second Affiliated Hospital of Anhui Medical University. Hefei, China.

^7^Center for Scientific Research, Anhui Medical University. Hefei, China.

^8^Center for Computational Integrative Biology (CCIB), Massachusetts General Hospital (MGH), Harvard Medical Colleague, MA, Boston, USA.

**Correspondence**

*Guolin Zhou,* *CCIB at MGH of Harvard Medical Colleague, MA, Boston, USA. E-mail:* [*GZHOU@CCIB.MGH.HARVARD.EDU*](mailto:GZHOU@CCIB.MGH.HARVARD.EDU)

*Haisheng Zhou, Department of Biochemistry, Anhui Medical University. Hefei, China. E-mail:* [*haishengs@ahmu.edu.cn*](mailto:haishengs@ahmu.edu.cn)

**Supplementary material and Methods**

**Creation of conditional knock out *Gpr107* mice**

Two targeting vectors (TV1 and TV2) were designed to label GPR107 and conditionally knock out *Gpr107.* To label the GPR107 protein expression and construct the conditional *Gpr107^hgfp_floxneo^* allele, we have made two targeting vectors, one that contains H2B-GFP fragment and Bxb1 attB and attP flanked puro-tk site, another that contains two *Loxp* sites flanking the entire exons 3 and 4 and neomycin site flanked by two Frt sites. The first targeting vector (TV1) was inserted upstream of the start codon ATG and fuses histone 2B-gfp (H2B-GFP, hgfp) with GPR107, and the second targeting vector (TV2) modifies exons 3 and 4 flanked by two *loxp* sites and the selection markers located before the second loxp site goes into intron between exon 4 and 5 (Supplementary Figure S2a). A bacterial artificial chromosome (BAC) containing the entire *Gpr107* was sequentially modified by those two targeting vectors by homologous recombination in *E. Coli* to generating the recombinant BACs (rec BAC), CKO *Gpr107* and CKO tag *Gpr107*. The two steps of recombination in E.Coli were identified by PCR analysis by using primer pairs P1-P8 indicated in the Supplementary Figure S2a. The PCR results showed that six kanamycin resistant clones carried the right recombination after first recombination, and two out of six trimethoprim resistant clones carries the right recombination after second recombination (Supplementary Figure S2b). The correctly recombinant BAC DNAs were validated further through changes in the digestion map of EcoR1 digestion (Supplementary Figure S2c). The resulting BAC DNA was used to target mouse W4/129S6 embryonic stem cells (Taconic) to create conditional *Gpr107^hgfp_floxneo^* allele (method described before)^1^. Over ten ES targeted ES clones were identified by multiplex ligation-dependent probe amplification (MLPA)[1] using the probes indicated in Supplementary Figure S2d. Wild-type peaks, WT1, WT2 and WT3 of the target ES cells decreased about half compared to that of wild type and non-target ES cells (Supplementary Figure S2e). The puro-TK cassette was deleted by transient expression of *Bxb1* integrase in correctly targeted clones. The resultant ganciclovir (GCV)-resistant cells were tested using PCR analysis with a pair of primers P9 and P10. PCR products were sequenced to confirm the deletion events (data not shown).

Chimeric mice were generated by injecting C57BL/6 blastocysts with 8–12 cells that had been derived from the above engineered embryonic stem lines according to the standard protocol. Seven male chimeric animals were produced. Those chimeric animals were mated with flp mice from Jax (B6.129S4-Gt (ROSA)26Sortm2(FLP*) Sor/J, Strain #:012930) to remove neomycin cassette on genomic DNA, two of which transmitted the conditional *Gpr107^hgfp_flox^* mutation into the next generation. Heterozygous mice lack of neomycin cassette and flp-free were mated with *Cre* mice from Jax (B6.129-Gt (ROSA)26Sortm1(cre/ERT2) Tyj/J, Strain #:008463), and then mating was initiated to generate homozygous mutants for conditional *Gpr107^hgfp_flox^* and *Cre* sites. The heterozygous progeny of chimeric animals and homozygous progeny of heterozygous animals for conditional *Gpr107^hgfp_flox^* site were validated using MLPA (data not shown). Genotyping of *Flp* and *Cre* were conducted by following manufacture’s protocol.

The resulting homologous animals for conditional *Gpr107^hgfp_flox^* mutation and Cre sites were mated with heterozygous traditional *Gpr107* knock out line (*Gpr107^ko+/-^*) which is embryonic lethal for homologous animals (*Gpr107^ko-/-^*), described in the previous article[2]. The resulted off springs contain two types of genotypes *Gpr107^hgfp_flox+/-^* (one allele is wild type, another allele is conditional *gpr107*) and *Gpr107^hgfp_flox-/ko-^* (one allele is traditional *Gpr107* knock out, another allele is conditional *Gpr107^hgfp_flox^*) in half by half. All those animals contain Cre site (heterozygotes). The induction of Cre recombinase would lead to deletion of exons 3 and 4 of conditional allele in *Gpr107^hgfp_flox-/ko-^*animals and subsequent inactivation of GPR107 (lost of 83-127 aa of 551 aa) since another allele lacks *Gpr107*. The post induction animals were labelled as *Gpr107^cko-/ko-^*.

Genotypes of the mice bearing *Gpr107^hgfp_flox^* (before induction), *Gpr107^cko^* (post induction) and wild-type allele were determined by PCR analysis. The positions of three primers are illustrated in Supplementary Figure S2d. A 292bp fragment was amplified from wild-type allele, a 360bp fragment was amplified from *Gpr107^hgfp_flox^* allele and a 483bp fragment were amplified with deletion of exons 3 and 4 from *Gpr107^cko^* allele (Supplementary Figure S2f). The two types of animal genotypes described above, named WT mice and KO mice, were used for the late-stage phenotypic study of GPR107.

TV1 has trimethoprim resistance and TV2 has kanamycin resistance. The targeting BAC was constructed by sequential homologous recombination between two targeting vectors and BAC clone *RP23-361I14* in Escherichia coli, following general procedures that have been described elsewhere[3]. The targeting BAC was linearized at a single PI-SceI site and electroporated into mouse W4/129S6 embryonic stem cells (Taconic). Clones that were resistant to G418 (200 mg/ml) were screened using MLPA with multiplex MLPA probes (Supplementary Table 2). Over ten positive clones were identified out of four 96-well plates of clones tested. Two clones were transfected with an *Bxb1* integrase expression plasmid to delete the puro-tk cassette. The resultant ganciclovir (GCV)-resistant cells were tested by using PCR analysis with a pair of primers (shown in Supplementary Table 3). PCR products were sequenced to confirm the deletion events. Chimeric mice were generated by microinjecting C57BL/6 blastocysts with 8–12 cells that had been derived from the engineered embryonic stem lines. Those chimeric animals were mated with *flp* mice from Jax (B6.129S4-Gt (ROSA) 26Sortm2 (FLP*) Sor/J, Strain #:012930) to remove neomycin cassette on genomic DNA. two of which transmitted the conditional *Gpr107^hgfp_flox^* mutation into the next generation. Heterozygous mice lack of neomycin cassette and flp-free were mated with *Cre* mice from jax (B6.129-Gt (ROSA) 26Sortm1 (cre/ERT2) Tyj/J, Strain #:008463), and then mating was initiated to generate homozygous mutants for conditional *Gpr107^hgfp_flox^*and *Cre* sites. Two of chimeric animals transmitted the *Gpr107^hgfp_floxneo^* mutation into the next generation. In the way of mating, existence of *Flp* removed the neomycin cassette in the intron to create *Gpr107^hgfp_flox^*mutation in one allele. Heterozygous mice were maintained, and mating was initiated to generate homozygous mutants for both *Gpr107^hgfp_flox^* and *Cre* sites. The above described two types of genotypes of animals, named as WT mice and KO mice, were used for the late-stage phenotypic study of GPR107.

All mice used in this study were derived from a congenic C57BL/6 background. Three-week-old WT mice (*Gpr107^hgfp_flox^* mice) were administered Tamoxifen (10 mg/kg) to induce knockout of *Gpr107* (named KO mice). The genomic DNA of the mice tails were subjected to PCR analysis for determining their genotypes.

The blood glucose levels in the tail vein were measured on a weekly basis using a glucometer (Roche). Values exceeding 16.7 mmol/L were considered an indication of a successful creation of the model. The mice were then maintained on a normal diet for a period of 12 weeks. Meanwhile, the body weight of mice was measured and recorded on a weekly basis. By the end of the 20-week experimental period, we collected 24-hour urine samples prior to euthanizing the mice. The kidney tissues were then separated and either stored in liquid nitrogen for protein extraction or fixed with paraformaldehyde. All experimental procedures involving animals were approved by the Institutional Animal Care Committee of Anhui Medical University.

**Real-time PCR**

Total RNA was extracted from cells using the TRIzol^TM^ reagent. Reverse transcription was performed to generate the first strand cDNA using 1 µg of RNA with the Evo M-MLV RT Premix Kit. A total of 1 µg of cDNA products was amplified in a 10 µl reaction system with SYBR Green Premix Pro Taq HS qPCR Kit in a Real Time PCR System (Roche). The cycling program was 95℃ for 30 s followed by 40 cycles of 95℃ for 10 s, 60℃ for 30 s. The primer sequences used for amplification are presented in Supplementary Table 4. Relative gene expression was obtained after normalization with GAPDH and followed by comparison with the control groups.

**Western Blot**

Total proteins were extracted from mice kidney tissues or HPC using RIPA buffer. Equal amounts of protein were separated by sodium dodecyl sulfate polyacrylamide gel electrophoresis (SDS-PAGE). Then samples were transferred to PVDF membrane and incubated in 5% milk. After blocking with 5% milk, the membrane was incubated with primary antibodies overnight and then washed with TBST and incubated with anti-mouse (1:2000), or anti-rabbit IgG (1:2000) horseradish peroxidase-conjugated secondary antibodies. Proteins were visualized using the Western Bright ECL kit (Advansta). Target proteins were quantified using the image J software.

**RNA sequencing**

The construction and sequencing of the library was carried out by Lianchuan Biotechnology Corporation according to manufacturer's requirements (Hangzhou, China).

**Co- immunoprecipitation (Co-IP)**

Co-IP was carried out using a Pierce Crosslink Magnetic Co-IP kit (Thermo Fisher). Briefly, the expression vectors, including the Flag-tagged GPR107 (pCMV-Tag2B-GPR107), His-tagged AT1R (pcDNA3.1-His-C-AT1R), and HA-tagged clathrin (pcDNA3.1-HA-N-Clathrin), were transiently transfected in HPC by Lipofectamine 3000. After 48 hours of transfection, the cells were lysed, and the protein concentrations were measured. Then 500 µg of protein in 500 µl of supernatant was subjected to overnight incubation at 4 ℃ on a rotator with 5 µg of anti-GPR107, anti-Flag, anti-His, anti-HA, or anti-IgG antibodies immobilized on beads. The beads were washed to remove non-bound materials using a low-pH elution buffer. The precipitated was separated by SDS-PAGE and detected by immunoblotting.

**Intracellular Ca^2+^ activity, Ca^2+^ concentration and Ca^2+^ Imaging**

Intracellular Ca^2+^ activities, Ca^2+^ concentrations, and Ca^2+^ Imaging were performed following the manufacturer’s protocols. Briefly, HPC cells were cultured in medium with NG or HG for varying durations based on the specific objective of the assay. The medium was removed and washed with Hank’s balanced salt solution (Sigma) for 3 times. Then HPC cells were co-cultured in the medium with Fluo-4 AM at 37 ℃ for 30 minutes. Subsequently, replaced with new medium without Fluo-4 AM for an additional 20 minutes at 37 °C. Intracellular Ca^2+^ activities were measured by Ca^2+^ imaging system (OLYMPUS). After the fluorescence signal was recorded and stabilized, the cells were treated with Ang-II (100 μM). The concentrations of Ca^2+^ was measured by CytoFLEX Analysis flow cytometer (Beckman). The fluorescence imaging was obtained by fluorescence microscope.

**Supplementary References**

1. Langerak P, Nygren AO, Schouten JP, Jacobs H. Rapid and quantitative detection of homologous and non-homologous recombination events using three oligonucleotide MLPA. Nucleic Acids Res. 2005;33(22):e188. <https://doi.org/10.1093/nar/gni187>

2. Zhou GL, Na SY, Niedra R, Seed B. Deficits in receptor-mediated endocytosis and recycling in cells from mice with Gpr107 locus disruption. J Cell Sci. 2014;127(Pt 18):3916-27. <https://doi.org/10.1242/jcs.135269>

3. Yang Y, Seed B. Site-specific gene targeting in mouse embryonic stem cells with intact bacterial artificial chromosomes. Nat Biotechnol. 2003;21(4):447-51. <https://doi.org/10.1038/nbt803>

**
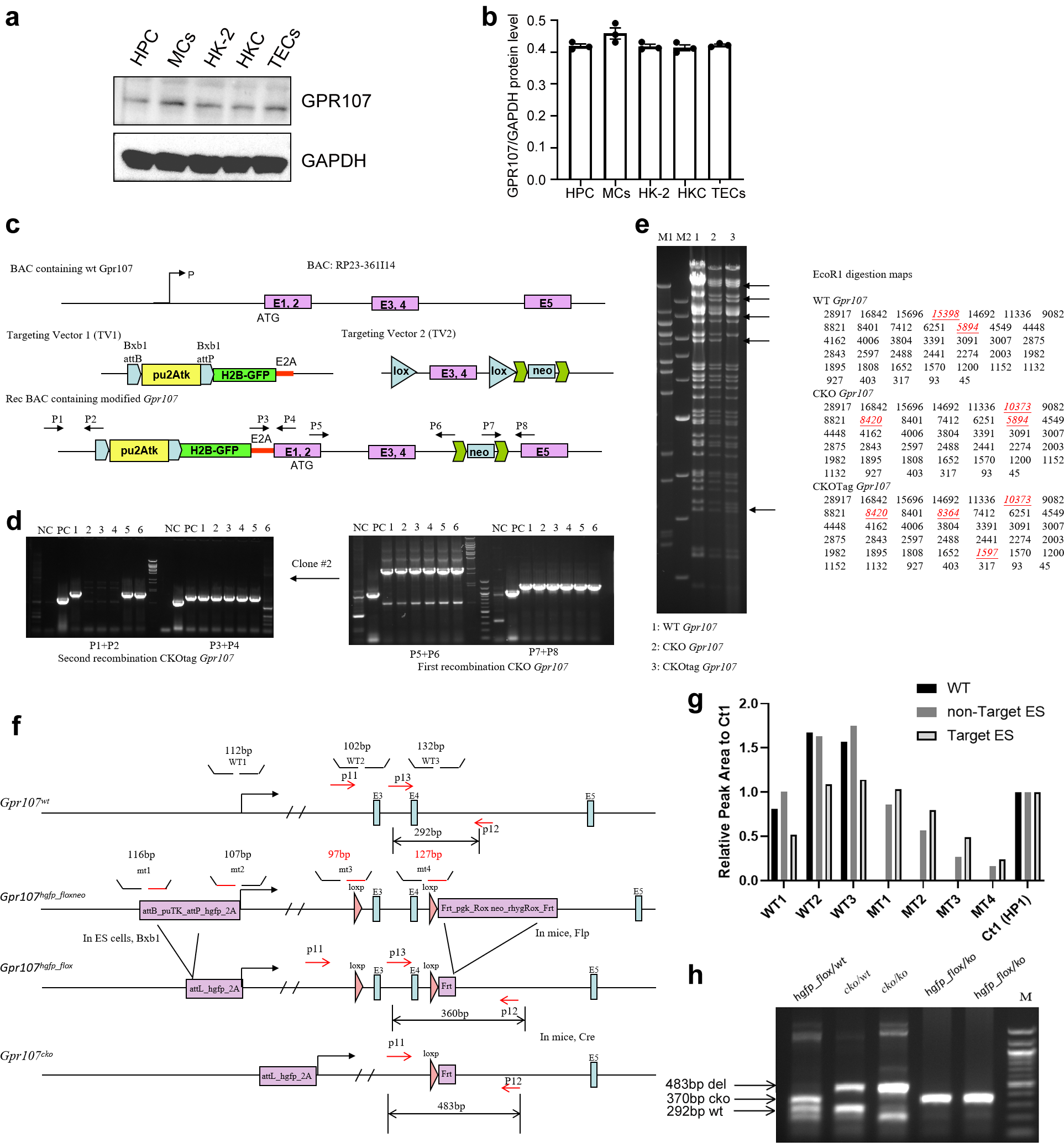
**

**Supplementary Fig. S1** Expression of GPR107 in different renal cell lines and the generation of conditional knock out *Gpr107* mice


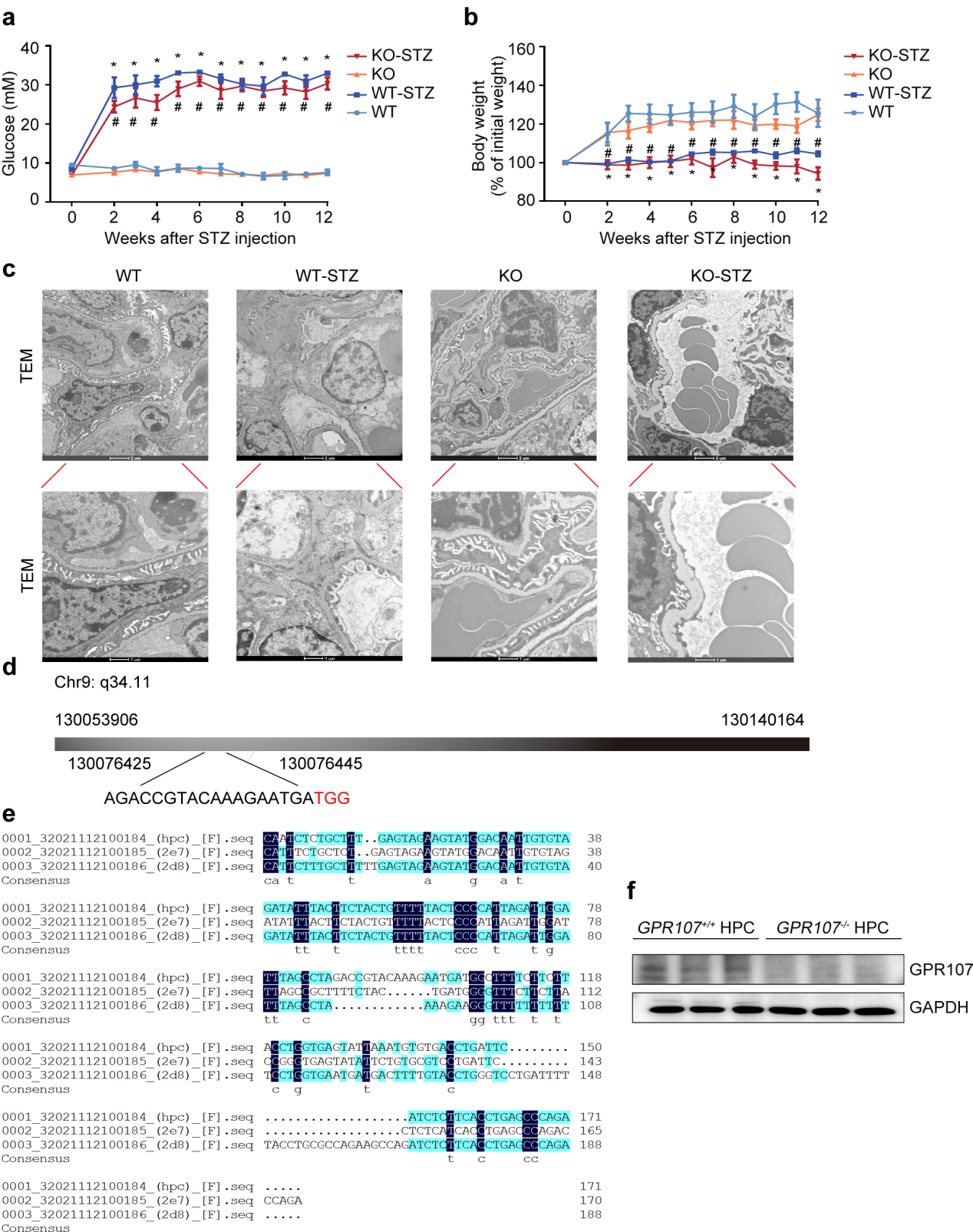


**Supplementary Fig. S2** The blood glucose levels and body weights of mice and identification of *GPR107*-deficient HPC

**
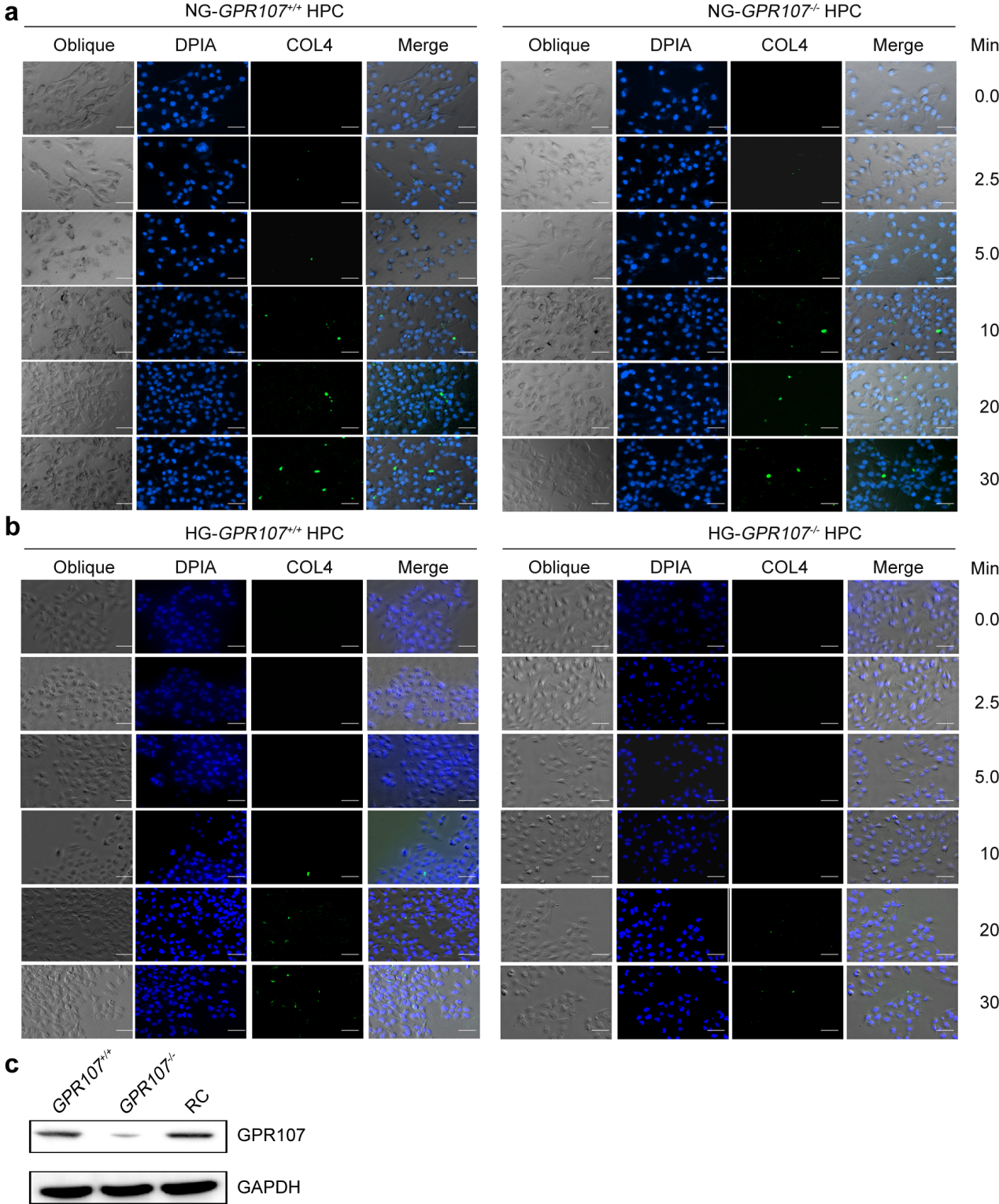
**

**Supplementary Fig. S3** GPR107 deletion decreases endocytosis of COL4 within podocytes and identification of rescue of *GPR107*^-/-^ HPC


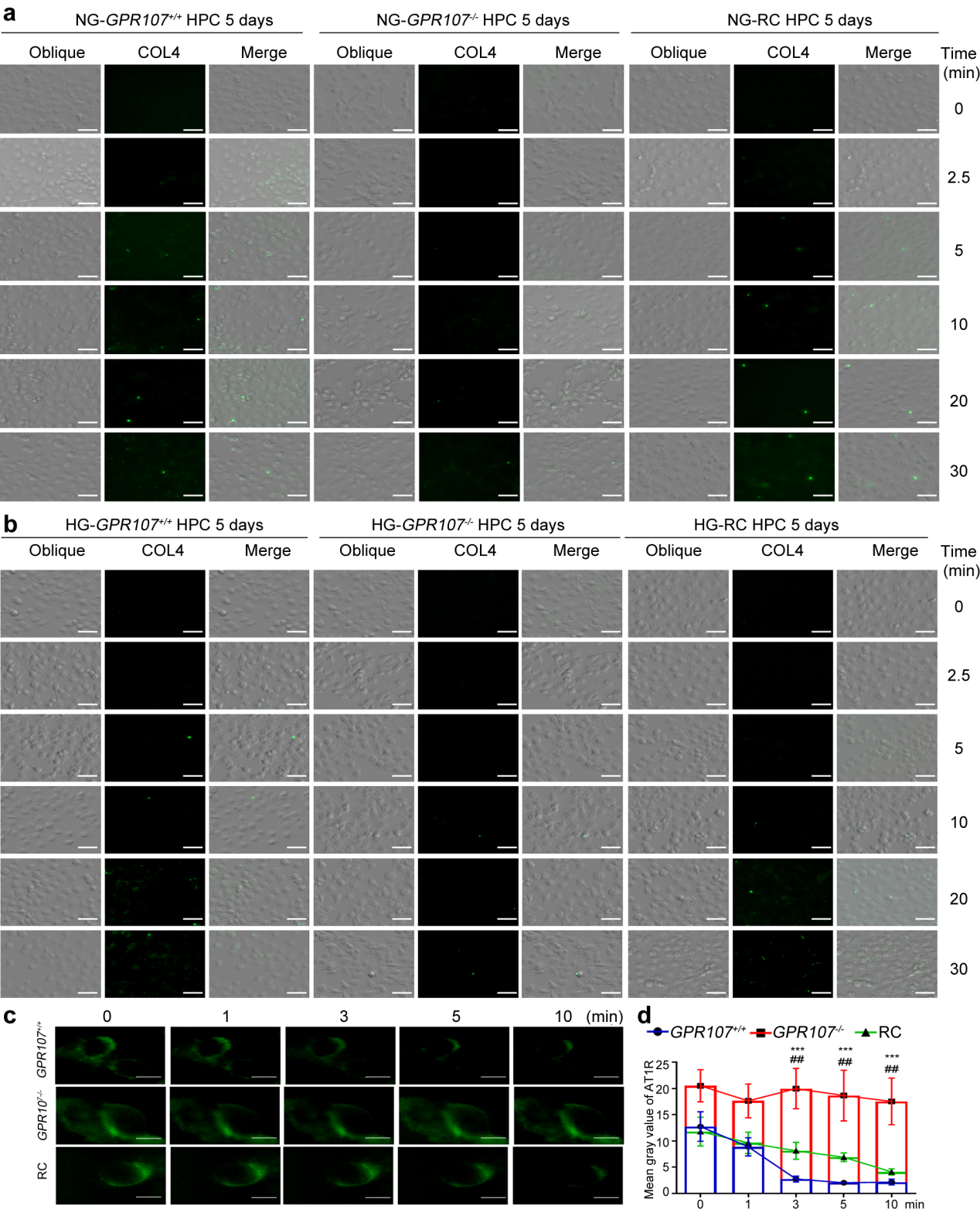


**Supplementary Fig. S4** The endocytosis of COL4 and AT1R in podocytes

**
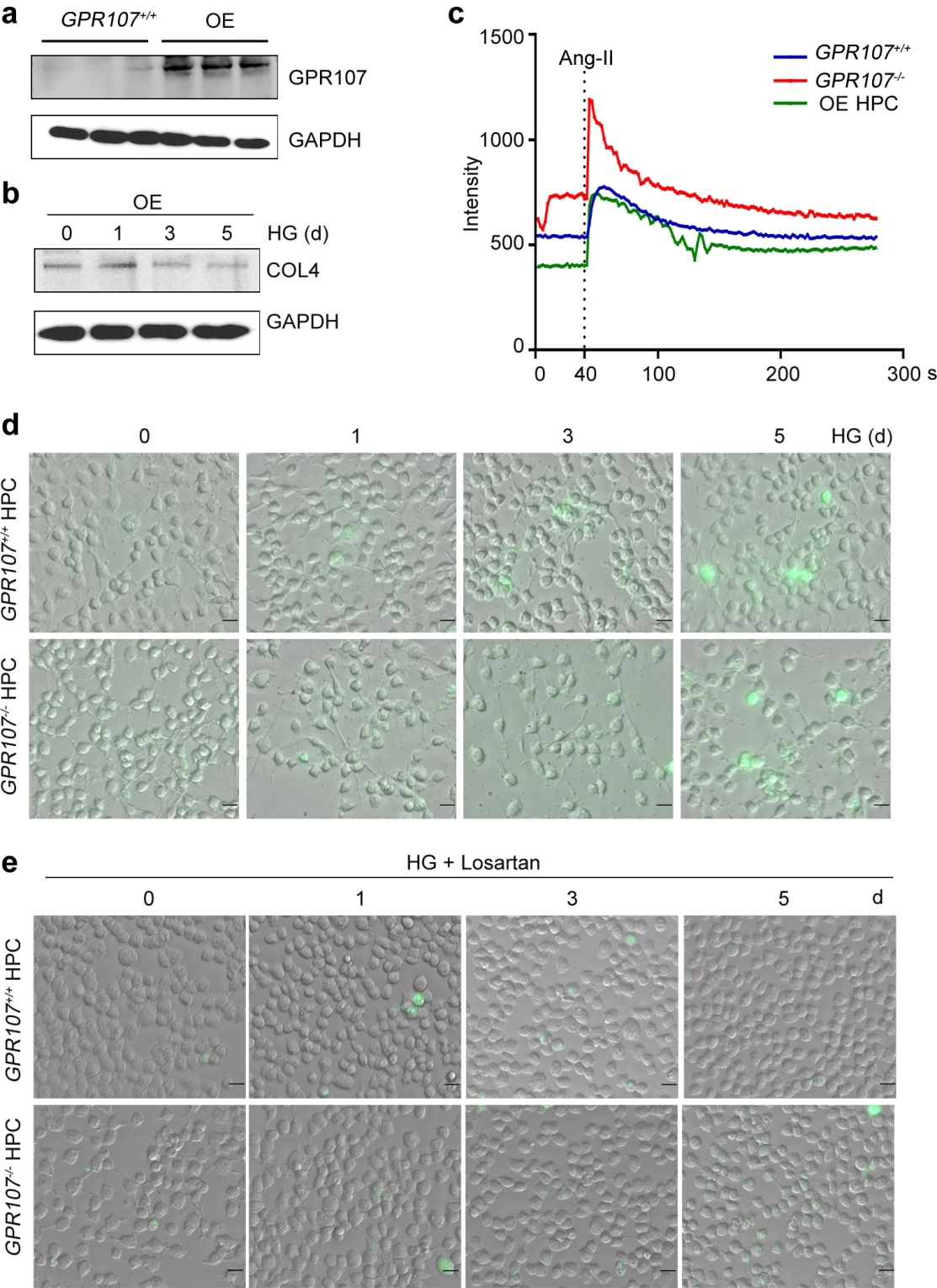
**

**Supplementary Fig. S5** The responses to Ang-II-induced calcium influxes or high glucose-induced Ca^2+^ concentration and upregulation of COL4 in OE HPC

**
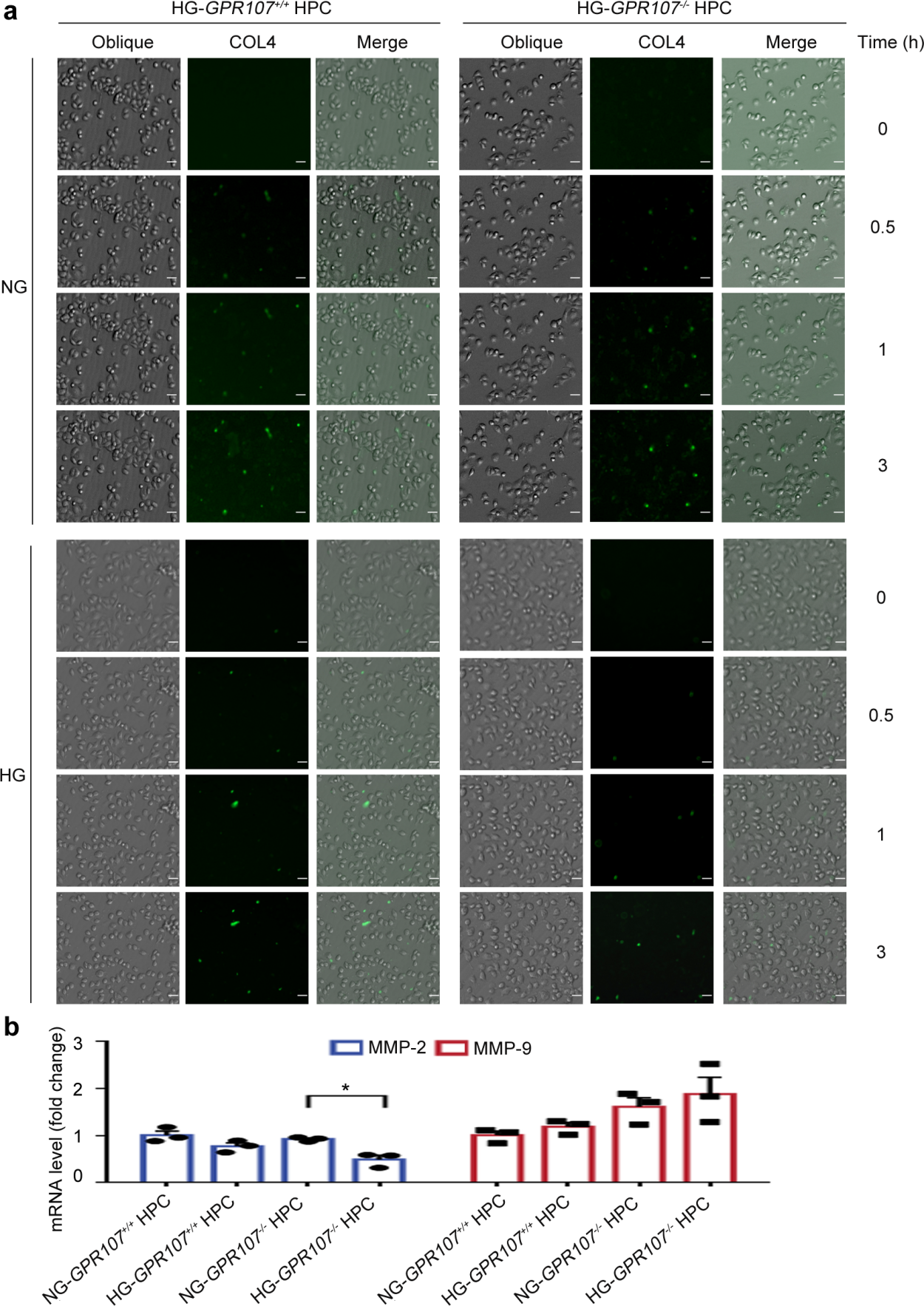
**

**Supplementary Fig. S6** High glucose inhibits the degradation of COL4 in the extracellular matrix of podocytes and RT-qPCR analysis for the expression levels of MMP-2 and MMP-9 in HPC

**Supplementary Figure legends**

**Supplementary Fig. S1 Expression of GPR107 in different renal cell lines and the generation of conditional knock out *Gpr107* mice**

**(a, b)** Western blotting and analysis for GPR107 expression in the HPC, MCs, HK-2, HKC and PTC. **(c)** Schematic figure of modification of BAC to generate conditional *Gpr107* knock out mice. **(d)** Results of PCR analysis of six recombinant clones for two steps of recombination. **(e)** Recombinant BAC validation by EcoR1 digestion. Three different versions of BAC, wild-type (WT), CKO (after recombination 1) and CKOtag *Gpr107* (after recombination 2) were digested by EcoR1 and run on 1% agrose gel. The arrows point the size-altered bands (Italic and underlined in maps) compared to wild-type BAC. **(f)** Schematic figure of targeting *Gpr107* in ES cells and genotyping for evaluating conditional knockout mice. **(g)** Representative MLPA data of WT, non-targeting ES and target ES cell clones. **(h)** Representative genotype data by PCR using primer P11, P12, P13 (shown in D). HPC, human podocytes. MCs, mesangial cells. HK-2, proximal tubular epithelial cells. HKC, distal tubular epithelial cells. TECs, primary cultured tubular epithelial cells. NC, negative control without recombinant DNA. PC, positive control to amplify the homology arms. 1-6 indicates different clones. M indicates DNA marker.

**Supplementary Fig. S2 The blood glucose levels and body weights of mice and identification of *GPR107*-deficient HPC**

**(a, b)** The blood glucose and body weights levels of mice. * *P*<0.001 WT-STZ compared with the WT mice. ^#^ *P*<0.001 KO-STZ compared with the KO mice. **(c)** Representative images of transmission electronic microscopy. Original magnification × 3200, bar = 2 μm. Original magnification × 6400, bar = 1 μm. **(d)** Schematic diagram for deletion of *GPR107* gene in the HPC. **(e)** DNA sequencing analysis of two single cell clones with *GPR107* mutation. **(f)** Western blotting analysis for GPR107 expression in the 2D8 HPC clone. Lane 1-3, wild type HPC; lane 4-6, the 2D8 HPC clone. The 2D8 cell line was used as the *GPR107^-/-^* HPC in the following experiments in this study. WT, wild type. KO, knockout. STZ, streptozotocin. WT, KO, and WT-STZ mice, n=4. KO-STZ mice, n=8. HPC, human podocytes.

**Supplementary Fig. S3 GPR107 deletion decreases endocytosis of COL4 within podocytes and identification of rescue of *GPR107*^-/-^ HPC**

**(a, b)** The *GPR107*^+/+^ and *GPR107*^-/-^ HPC cells were co-cultured with the quenched fluorescent substrate DQ-COL4 for different time points (0, 2.5, 5, 10, 20, and 30 min) at 37℃ under the NG or HG. Original magnification ×200, bar =20 μm. **(c)** Western blotting analysis for GPR107 expression in the *GPR107*^+/+^ HPC, *GPR107*^-/-^ HPC and RC HPC. HPC, human podocytes. RC, rescue of *GPR107*^-/-^ HPC.

**Supplementary Fig. S4 The endocytosis of COL4 and AT1R in podocytes**

**(a, b)** The *GPR107^+/+^*, *GPR107^-/-^* and RC HPC cells were co-cultured with the quenched fluorescent substrate DQ-COL4 for different time points (0, 2.5, 5, 10, 20, and 30 min) at 37℃ under the NG or HG. Original magnification ×100, bar =50 μm. **(c, d)** Analyzing the distribution of AT1R on the cell membrane. Green fluorescence signals were observed in both the *GPR107^+/+^*, *GPR107^-/-^* and RC HPC cells that were transfected with the GFP-tagged AT1R, and then treated with Ang-II (100 μM) for 1, 3, 5, and 10 minutes. RC, rescue of *GPR107*^-/-^ HPC. *** *P*<0.001, compared with *GPR107^+/+^* HPC. ^##^ *P*<0.01, compared with RC HPC. HPC, human podocytes. RC, rescue of *GPR107*^-/-^ HPC.

**Supplementary Fig. S5 The responses to Ang-II-induced calcium influxes or high glucose-induced Ca^2+^ concentration and upregulation of COL4 in OE HPC**

**(a)** Western blotting analysis for GPR107 expression in HPC and OE HPC. **(b)** Western blotting analysis for COL4 expression in the OE HPC after exposure to high glucose for 0, 1, 3, 5 days. **(c)** Representative images of Ca^2+^ influxes in HPC induced by Ang-II. **(d, e)** The fluorescence images of HPC under HG with or without Losartan (10 μM). The images were captured using a fluorescence inverted microscope. Original magnification ×200, bar =20 μm).

HPC, human podocytes. OE HPC, HPC overexpression of GPR107.

**Supplementary Fig. S6 High glucose inhibits the degradation of COL4 in the extracellular matrix of podocytes and RT-qPCR analysis for the expression levels of MMP-2 and MMP-9 in HPC**

**(a)** Green fluorescent degradation products of DQ-COL4 in the ECM of HPC. Images of the live cells were captured at 0.5, 1, 2, and 3 hours. Original magnification ×200, bar =20 μm. HPC, human podocytes. **(b)** RT-qPCR analysis was performed to detect the expression levels of MMP-2 and MMP-9. * *P*<0.05. HPC, human podocytes.

**Table S1** Antibodies Table (used in current study)

| **Target antigen** | **Vendor or Source** | **Catalog #** | **Working  concentration** |
| --- | --- | --- | --- |
| Rabbit anti-GPR107 | Proteintech | 25076-1-AP | 1:1000 (WB) / 1:50 (IHC) |
| Mouse anti-ATIR | Snata cruz | SC-515884 | 1:1000 (WB) / 1:100 (IHC) |
| Rabbit anti-ATIR | Proteintech | 25343-1-AP | 1:1000 (WB) / 1:50 (IP) |
| Mouse anti-clathrin | Snata cruz | SC-12734 | 1:1000 (WB) |
| Mouse anti-MMP-2 | Snata cruz | SC-13594 | 1:500 (WB) / 1:100 (IF) |
| Rabbit anti-MMP-2 | Proteintech | 10373-2-AP | 1:100 (IHC) |
| Rabbit anti-COL4 | Huabio | HA500197 | 1:1000 (WB) / 1:100 (IF) |
| Rabbit anti-COL4 | Novus | NB120-6586 | 1:100 (IHC) |
| DYKDDDDK Tag (D6W5B) Rabbit | Cell Signaling Technology | 14793 | 1:1000 (WB) / 1:50 (IP) |
| Rabbit anti-CREB | Cell Signaling Technology | 9197 | 1:1000 (WB) |
| Rabbit anti-Phospho-CREB | Cell Signaling Technology | 9198 | 1:1000 (WB) |
| Rabbit anti-GAPDH | Huabio | ET1601-4 | 1:5000 (WB) |
| Mouse anti-His | Abmart | M30111S | 1:1000 (WB) / 1:50 (IP) |
| Mouse anti-HA | Abmart | M20003S | 1:1000 (WB) / 1:50 (IP) |
| anti-rabbit IgG | Beyotime | A7016 | 1:50 (IP) |
| anti-mouse IgG | Beyotime | A7028 | 1:50 (IP) |
| Anti-mouse IgG, HRP linked Antibody | Cell Signaling Technology | 7076 | 1:5000 |
| Anti-rabbit IgG, HRP linked Antibody | Cell Signaling Technology | 7074 | 1:5000 |
| Anti-mouse IgG (H+L), F(ab')2 Fragment (Alexa Fluor® 488 Conjugate) | Cell Signaling Technology | 4408S | 1:500 |
| Anti-rabbit IgG (H+L), F(ab')2 Fragment (Alexa Fluor® 594 Conjugate) | Cell Signaling Technology | 8889S | 1:500 |

**Table S2** Primer sequences for PCR

| **Name** | **Sequence** |
| --- | --- |
| P1 | TAGGAATCTGATTCTGTCATAGGT |
| P2 | AGCGGGGCTGCTAAAGCGCATGCTC |
| P3 | CGAGCTGTACAAGACTAGTGGCTC |
| P4 | CTGTGTCCAGATCCGCAACTTCTTG |
| P5 | GACATCAACCCCAATCTCTGTAGGA |
| P6 | TCGATCAGAAACTTCTCGACAGACG |
| P7 | GGGCTGCTAAAGCGCATGCTCCA |
| P8 | CTCGCTGACCTCCATATGCTCTGAC |
| P9 | GAGGTGGTCCGCTCAGGTCCCTGC |
| P10 | TGGACCTGCTTCAGAACCTTGTACA |
| P11 | CTTGAGCAGCTGTGTCCTTCT |
| P12 | GCCTAAGGCTGACCTGAATC |
| P13 | CATGTCTTCTGTCACTCTTGTCATC |

**Table S3** Probe sequences of MLPA

| **Products** | **Name** | **Sequence** | **Size (bp)** |
| --- | --- | --- | --- |
| WT1 | wt1-Lig | GGGTTCCCTAAGGGTTGGAGGTGGGAGAGGAAGCAGCTGGTGATGCTGGAACAAAC | 112 |
|  | wt1-NPK | ATGGCCGTCCCGGTACCCCTCGGCCGCTTCGGCCCTAGATTGGATCTTGCTGGCGC |  |
| MT1 | mut1-NPK | TCGGCCGGCTTGTCGACGACGGCGGTCTCCGTCGTCACCTAGATTGGATCTTGCTGGCGC | 116 |
| MT2 | mut2-lig | GGGTTCCCTAAGGGTTGGAGCGATGTTGAAAGTAACCCCGGTCCTGGATCC | 107 |
|  | mut2-NPK | GCCGTCCCGGTACCCCTCGGCCGCTTCGGCTCCCCTAGATTGGATCTTGCTGGCGC |  |
| WT2 | wt2-Lig | GGGTTCCCTAAGGGTTGGACAAGGGCAGAGGATAGTGTGAGGGACTTA | 102 |
|  | wt2-NPK | GTCTGTCCTCTCTTTGCTGCCCTCAGATCGGCCTAGATTGGATCTTGCTGGCGC |  |
| MT3 | mut3-NPK | ATAACTTCGTATAAAGTATCCTATACCCTAGATTGGATCTTGCTGGCGC | 97 |
| WT3 | wt3-Lig | GGGTTCCCTAAGGGTTGGACCGACAGAGGACAATCCAAATGACTGACTGCAGCACAGTGGAGGAAAGGAAGAGGGGTT | 132 |
|  | wt3-NPK | TGCACGCCATGGAAGGACGGATTCAGGTCAGCCTAGATTGGATCTTGCTGGCGC |  |
| MT4 | mut4-NPK | ATAACTTCGTATAAAGTATCCTATACCCTAGATTGGATCTTGCTGGCGC | 127 |
| MT5 | mut5-lig | GGGTTCCCTAAGGGTTGGACCAATTATTTAAAGTTACAATTGAAGGATCTGAAGTTCCTATACTTTCTAGAGAATAGGAACTTCGGATCC | 144 |

**Table S4** Primer sequences for real-time PCR

| **Gene** | **Forward** | **Reverse** |
| --- | --- | --- |
| Human_COL4A1 | 5'-CTAATGTCACAACATGGTGCTAC-3' | 5'-GCAGGGTGTGTTAGTTACGC-3' |
| Human_MMP-2 | 5'-TACAGGATCATTGGCTACACACC-3' | 5'-GGTCACATCGCTCCAGACT-3' |
| Human_MMP-9 | 5'-GACGTCTTCCAGTACCGA-3' | 5'-CTCAGGGCACTGCAGGAT-3' |
| Human_CALM3 | 5'-GACCATTGACTTCCCGGAGTT-3' | 5'-GATGTAGCCATTCCCATCCTTG-3' |
| Human_CAMK4 | 5'-GCCTCGTCCCGGATTACTG-3' | 5'-TCCCCTTCTGTTTGCATCTGT-3' |
| Human_GAPDH | 5'-TCACTGCCACTCAGAAGACTGT-3' | 5'-CGTTCAGCTCTAGGATGACCTT-3' |
